# Boronic Acid‐Mediated Activity Control of Split 10–23 DNAzymes

**DOI:** 10.1002/chem.202004227

**Published:** 2020-12-15

**Authors:** Mégane Debiais, Amandine Lelievre, Jean‐Jacques Vasseur, Sabine Müller, Michael Smietana

**Affiliations:** ^1^ Institut des Biomolécules Max Mousseron Université de Montpellier CNRS ENSCM Place Eugène Bataillon 34095 Montpellier France; ^2^ University Greifswald Institute for Biochemistry Felix-Hausdorff-Strasse 4 17487 Greifswald Germany

**Keywords:** autoligation, boronic acids, DNA, oligonucleotides, RNA

## Abstract

The 10–23 DNAzyme is an artificially developed Mg^2+^‐dependent catalytic oligonucleotide that can cleave an RNA substrate in a sequence‐specific fashion. In this study, new split 10–23 DNAzymes made of two nonfunctional fragments, one of which carries a boronic acid group at its 5′ end, while the other has a ribonucleotide at its 3′ end, were designed. Herein it is demonstrated that the addition of Mg^2+^ ions leads to assembly of the fragments, which in turn induces the formation of a new boronate internucleoside linkage that restores the DNAzyme activity. A systematic evaluation identified the best‐performing system. The results highlight key features for efficient control of DNAzyme activity through the formation of boronate linkages.

## Graphical abstract

**Split, join, activate**: Split DNAzymes made of two or more short nucleic acid strands are emerging as novel tools for biosensing applications. Herein, it is reported that the activity of 10–23 DNAzyme that was split into two nonfunctional fragments can be restored by formation of a boronate internucleoside linkage.

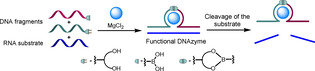

## Introduction

The spontaneous and reversible reaction between boronic acids and *cis*‐diols leading to the formation of cyclic boronate esters has been comprehensively investigated^[1]^ and has found widespread use for biomedical,^[2]^ sensing,^[3]^ materials,^[4]^ and supramolecular applications.^[5]^ This reaction is notably characterized by the ability of boronic acids to operate in aqueous media, and by the reactivity of the boron atom, which can be either sp^2^‐ or sp^3^‐hybridized depending on the addition of a Lewis base.^[6]^

**Figure 1:**
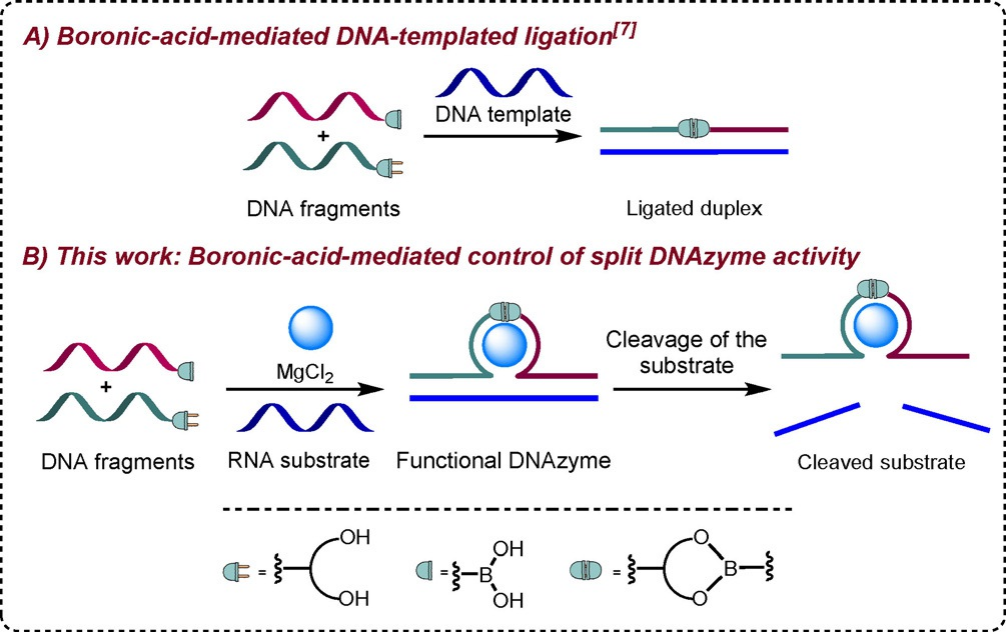
Schematic representation of boronic acid‐mediated assembly of nucleic acids.

In this context, we recently developed reversible DNA‐ and RNA‐templated boronate‐formation systems operating through the reaction of two oligonucleotide fragments, one of which has a boronic acid group at its 5′ end, and the other a ribonucleotide at its 3′ end (Figure 1 A).^[7]^ While boronate esters are known to be sensitive to water, the presence of the template increases significantly the effective molarity of the two oligonucleotide fragments and facilitates the reaction.^[8]^ The resulting strand therefore differs from natural DNA by having a boronate internucleoside linkage instead of a phosphodiester. Inspired by these results we decided to evaluate whether the formation of boronate esters at key positions of engineered split DNAzymes could be triggered by an effector and subsequently be used to control their activity (Figure 1 B).

Like ribozymes, DNAzymes are capable of supporting a wide variety of reactions.^[9]^ Yet, the most prominent and best studied representatives of the DNAzyme family are RNA‐cleaving DNAzymes.^[10]^ Composed of a catalytic core and substrate‐recognizing domains, RNA‐cleaving DNAzymes catalyze the phosphodiester bond cleavage of their RNA substrate with the aid of a divalent cation. Among them, the 10–23 DNAzyme, which was named after the round and clone numbers of its respective in vitro selection, has been the most widely studied RNA‐cleaving DNAzyme.^[11]^ Its catalytic core is composed of a loop flanked by two complementary substrate‐recognition domains. On binding the substrate, which is a full oligoribonucleotide or an oligodeoxyribonucleotide with a single ribonucleotide at the cleavage site, the catalytically competent structure is formed in the presence of magnesium ions, followed by cleavage of a specific phosphodiester bond.^[12]^

The 10–23 DNAzyme catalytic core has been well studied through base or phosphate substitutions and deletions. The G_9_–G_14_ and C_21_–G_22_ regions were demonstrated to be directly involved in forming the catalytic site.^[13]^

Since DNAzymes are easy to synthesize and to modify, many studies have been devoted to improve their stability and catalytic properties.[^13d^, ^14^] In this context split systems, which rely on dividing a DNAzyme into a series of two or more independent fragments that are able to assemble in the presence of a specific target, are emerging as novel biosensing tools for the modulation of DNAzyme activity with high spatial and temporal resolution.^[15]^

Although split systems are yet easier to synthesize and carry fewer negative charges per strand, the design of efficient split systems can be a challenging task. The choice of the split site and the resulting stability and efficiency of the split DNAzyme–substrate complex are key issues that need to be considered, in particular when application of the split system in diagnostics or therapy is planned. Most of the systems rely solely on the target‐driven assembly of the fragments, which, however, often results in unstable and/or poorly active systems. This drawback can be counterbalanced by the use of fragments modified with chemical functionalities that upon addition of the target are brought into close proximity to allow covalent end joining of the fragments. Ideally, this reaction occurs without prior chemical activation. Therefore, fragment design and experimental verification of the activity of the assembled structure are required for the development of split DNAzymes.

## Results

**Figure 2:**
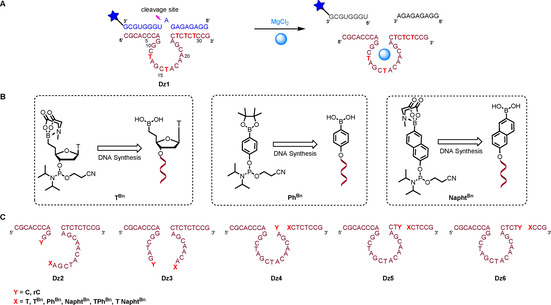
A) Secondary structure of wild‐type 10–23 DNAzyme (**Dz1**). The pink arrowhead indicates the cleavage site and the blue star represents the fluorescent dye ATTO680. Thymidine residues that have been replaced with structures shown in B) in individual experiments are highlighted in red. B) Phosphoramidite derivatives of boronic acid incorporated at various thymidine positions. C) Sequences of the split DNAzymes (**Dz2**–**Dz5**) employed in this study.

To achieve this control, the 10–23 DNAzyme was split into two fragments individually at all thymidine positions, located either in the catalytic loop or in one of the flanking arms. To monitor the performance of the different systems, the RNA substrate 5′‐GGAGAGAGAUGGGUGCG‐3′ was fluorescently labeled with ATTO680 at its 3′ extremity and the cleavage rates *k* of DNAzymes **Dz1**–**Dz6** were determined by quantification of the amount of cleaved RNA at eight different time points, analyzed by denaturing polyacrylamide gel electrophoresis (PAGE) on a LICOR DNA sequencer (Figure 2 A). Cleavage reactions were performed under single‐turnover conditions; hence, binding of the substrate and release of the cleavage products did not need to be considered.

The first fragment of each DNAzyme was modified at its 3′ extremity with a cytosine ribonucleotide, while the second fragment was modified at its 5′ extremity with a boronic acid moiety. Three phosphoramidite derivatives of boronic acid, namely **T^Bn^**, **Ph^Bn^**, and **Napht^Bn^**, were prepared and incorporated individually at all thymidine positions of the 10–23 DNAzyme (Figures 2 B and C). The phosphoramidite of **T^Bn^** was prepared according to our previously reported procedure.[^7d^, ^16^] Phosphitylation of commercially available 4‐hydroxyphenylboronic acid pinacol ester delivered the phosphoramidite derivative of **Ph^Bn^**, while the phosphoramidite of **Napht^Bn^** was obtained from 6‐hydroxynaphthalene‐2‐boronic acid after protection of the boronic acid as a MIDA ester and phosphitylation (Supporting Information). These derivatives were then incorporated at the 5′ end of the oligonucleotide sequences on an automated DNA synthesizer by using conventional phosphoramidite chemistry.

To further understand the effect of **Ph^Bn^** and **Napht^Bn^** modifications, we evaluated also the performance of split DNAzymes in which these moieties were added after the T_12_, T_16_, T_25_, T_27_, and T_29_ positions instead of replacing these final residues. In each case, the activity of the corresponding unmodified split DNAzymes was evaluated as control.

The Mg^2+^‐dependent catalytic ability was first evaluated on the parent **Dz1**, which showed a cleavage rate of *k*=0.364±0.073 min^−1^ and a percentage of RNA cleaved (*A)* of about 80 %, in accordance with previously reported data,^[17]^ while no reaction could be observed in the absence of Mg^2+^ ions (Table 1, entry 1 and Figure S19 in the Supporting Information).

**Table 1:** Observed rate constants and percentage of total RNA cleaved for 10–23 DNAzymes **Dz1** and split DNAzymes **Dz2**–**Dz6**.

Entry	DNAzyme	Y	X	*k* ^[a]^ [min^−1^]	*A* [%]
1	**Dz1**	–	–	0.364(±0.073)	75.6(±1.7)
					
2	**Dz2**	**C**	**T**	inactive	–
3		**rC**	**T^Bn^**	inactive	–
					
4	**Dz3**	**C**	**T**	0.039(±0.003)	79.6(±2.3)
5		**rC**	**T^Bn^**	0.127(±0.011)	82.7(±3.9)
6		**C**	**T^Bn^**	0.048(±0.007)	80.3(±4.2)
7		**rC**	**Napht^Bn^**	0.050(±0.005)	86.8(±3.2)
8		**rC**	**TNapht^Bn^**	0.068(±0.004)	82.1(±1.5)
					
9	**Dz4**	**C**	**T**	0.013(±0.002)	47.3(±5.1)
10		**rC**	**T^Bn^**	0.094(±0.017)	61.6(±3.1)
11		**rC**	**Ph^Bn^**	0.063(±0.011)	44.1(±2.6)
12		**rC**	**Napht^Bn^**	0.026(±0.001)	47.0(±1.1)
13		**rC**	**TPh^Bn^**	0.076(±0.013)	46.5(±2.5)
14		**rC**	**TNapht^Bn^**	0.021(±0.004)	42.5(±4.4)
					
15	**Dz5**	**C**	**T**	0.087(±0.012)	57.9(±2.2)
16		**rC**	**T^Bn^**	0.120(±0.024)	64.0(±3.2)
17		**rC**	**Ph^Bn^**	0.019(±0.003)	47.7(±4.2)
18		**rC**	**Napht^Bn^**	0.016(±0.006)	57.7(±12.7)
19		**rC**	**TPh^Bn^**	0.204(±0.058)	59.2(±3.0)
20		**rC**	**TNapht^Bn^**	0.033(±0.004)	53.0(±2.9)
					
21	**Dz6**	**C**	**T**	0.059(±0.007)	44.9(±1.8)
22		**rC**	**T^Bn^**	0.211(±0.037)	64.2(±2.0)
23		**rC**	**Ph^Bn^**	0.045(±0.005)	61.5(±2.4)
24		**rC**	**Napht^Bn^**	0.052(±0.008)	59.7(±3.3)
25		**rC**	**TPh^Bn^**	0.085(±0.013)	66.8(±3.1)
26		**rC**	**TNapht^Bn^**	0.057(±0.015)	70.8(±6.5)

[a] Observed rate constants of DNAzymes under single‐turnover conditions (2 h reaction at 25 °C, 50 mm Tris buffer (pH 8.6), 20 mm MgCl_2_, 20 nm of substrate, and 2 μm of each **Dz** fragment). First‐order rate constants *k* were obtained from curve fitting to [S]=*A*(1−e^−*kt*^), where [S] is the fraction of uncleaved substrate at time *t*, and *A* is the percentage of total RNA cleaved after 2 h of incubation.^[17]^

We started our study with the unmodified split DNAzyme **Dz2**, which showed no cleavage after 2 h of incubation. This result is coherent with previous studies, which demonstrated that nonbridging phosphate oxygen atoms located between C_11_ and T_12_ are involved in direct coordination of Mg^2+^ ions (Table 1, entry 2).^[13c]^ Consequently, it was not surprising that replacement of C_11_ by a ribocytosine and T_12_ by **T^Bn^** did not restore the activity (Table 1, entry 3). Interestingly, if the DNAzyme was split between C_15_ and T_16_ (**Dz3**), it was still active (*A*=79.6 %), although a sixfold reduction of the cleavage rate compared with **Dz1** was observed (*k*=0.039±0.003 min^−1^, Table 1, entry 4). However, replacing C_15_ by a ribocytosine and T_16_ by **T^Bn^** somewhat restored the activity (*k*=0.127±0.045 min^−1^, *A*=82.7 %), which suggests that the formation of a boronate internucleoside linkage influences the activity of the split DNAzyme (Table 1, entry 5). Interestingly, joining of the fragments occurs in a region in which flexibility is important. A control experiment with the fragment carrying the terminal C_15_ and the **T^Bn^**‐modified fragment confirmed this effect (Table 1, entry 6). Concerning the other evaluated **Dz3**‐based systems (Table 1, entries 7 and 8), very limited variations were observed compared with the unmodified split DNAzyme **Dz3**.

When the DNAzyme was split at the junction of the catalytic core and the flanking arm (**Dz4**), a remarkable decrease of product yield was observed (*A*=47.3 %; Table 1, entry 9), most probably due to the incapacity of the DNAzyme to adopt its active structure. Interestingly, the replacement of C_24_ by an rC residue and T_25_ by **T^Bn^** in **Dz4** restores the activity remarkably (Table 1, entry 10; *A*=61.6 %). All other modifications increase the cleavage rate by varying degrees, but product yields were similar to those of the parent split **Dz4** (Table 1, entries 11–14).

When the cut is positioned in the flanking arm between C_26_ and T_27_, the unmodified split DNAzyme **Dz5** (Table 1, entry 15) recovers some activity (*k*=0.087±0.012 min^−1^, *A*=57.9 %), although only three base pairs of the 5′ fragment are complementary to the RNA substrate. Here again, the formation of a boronate internucleoside linkage restores activity when C_26_ and T_27_ are replaced by a ribocytosine and **T^Bn^**, respectively (Table 1, entry 16; *k*=0.120±0.024 min^−1^, *A*=64.0 %). Surprisingly, in this specific configuration the highest cleavage rate is obtained when **Ph^Bn^** is added after T_27_. The activity of this split variant is nearly as high as that of the parent **Dz1** (Table 1, entry 19; *k*=0.204±0.058 min^−1^), although the percentage of total RNA cleavage is somewhat lower (*A*=59.2 %). The exact nature of the activity increase observed with X=**TPh^Bn^** in **Dz5** remains unclear. It is well known that the formation of boronate esters is favored for aromatic boronic acids compared with aliphatic ones.^[6]^ Hence, the rather high flexibility of the 3′‐terminal nucleotides of the 5′ fragment in **Dz5** might be counterbalanced by the rigidity of the aromatic boronate ester, formed when X=**TPh^Bn^**. By contrast, when **Ph^Bn^** replaces T_27_ (X=**Ph^Bn^**), the activity is noticeably reduced, most likely because the structure of the resulting DNAzyme becomes strained due to the missing nucleotide T_27_.

Lastly, split DNAzyme **Dz6** is characterized by a cut between C_28_ and T_29_, such that five base pairs at the 3′ terminus of the 5′ fragment remain for binding to the substrate. Surprisingly, activity of this split variant is somewhat lower than that of unmodified split DNAzyme **Dz5**, despite the two additional base pairs in **Dz6** (Table 1, entry 21; *k*=0.059±0.007 min^−1^, *A*=44.9 %). However, when C_28_ and T_29_ are replaced with **rC** and **T^Bn^**, respectively, a remarkable increase of the cleavage rate and product yield is observed (Table 1, entry 22, *k*=0.211±0.037 min^−1^, *A*=64.2 %). All other modifications also induced product yields higher than that of the unmodified split DNAzyme **Dz6**, albeit with lower cleavage rates (Table 1, entries 23–26).

Although the formation of boronate esters can be observed in neutral aqueous media, it is well known that the association between boronic acids and *cis*‐diols is favored at higher pH. This effect is explained by the formation of an hydroxyboronate complex resulting from rehybridization of the boron atom from sp^2^ to sp^3^ and a substantial release of angle strain around the boron center.^[18]^

**Figure 3:**
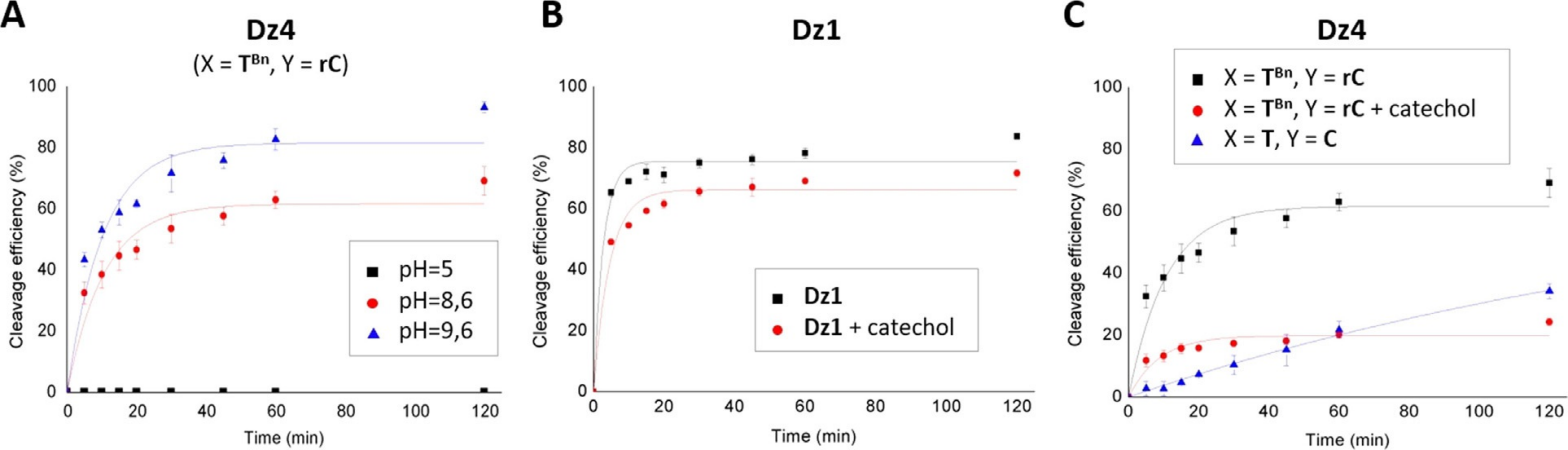
Time courses of cleavage reactions under single‐turnover conditions analyzed by a LICOR DNA sequencer. A) pH dependence of modified **Dz4**. B) Parent DNAzyme **Dz1** and C) split DNAzymes **Dz4** in the absence or presence of 1000 equiv of catechol.

Thus, we evaluated the cleavage activity of **Dz4** from pH 5.5 to pH 9.6. At pH 5.5 neither the parent **Dz1** nor its split **Dz4** analogues (X=**T** or **T^Bn^**, Y=**C** or **rC**) were found to be active. In the case of **Dz1**, increasing the pH value to 9.6 resulted in no significant difference in terms of cleavage rate or product yield (*A*=76 and 83 % at pH 8.6 and 9.6 respectively; Figures S1, S44–S45 in the Supporting Information). Similarly, the percentage of cleaved substrate obtained with the unmodified **Dz4** (X=**T**, Y=**C**) decreased from 58 to 47 % at pH 9.6 and 8.6 respectively (Figures S26, S46–S47 in the Supporting Information). However, in the presence of the **T^Bn^** modified split **Dz4** we were pleased to observe a ∼25 % increase of the percentage of product yield, when the pH value was raised to 9.6 (from 61.6 to 81.5 % at pH 8.6 and 9.6, respectively; Figure 3 A) thus nearly reaching the wild‐type activity after 2 h of incubation and this further demonstrates the ability of the noninvasive nature of borono‐modified systems to control DNAzyme activity through pH variations.

Finally, to provide definitive proof that the boronic acid–diol interaction controls the split‐DNAzyme activity, we performed a competition experiment in the presence of 1000 equiv of catechol. Catechol is known to form stable esters with boronic acid and thus would outcompete the 3′‐terminal diol in **Dz4**.^[19]^ As expected, the addition of catechol induced only a slight decrease of the percentage of cleaved substrate with **Dz1**, most likely as a consequence of nonspecific interaction of catechol with the DNA strands (from 76 to 66 %, Figure 3 B), whereas a dramatic loss of activity from 62 to 20 % was observed for **Dz4** (X=**T^Bn^**, Y=**rC**; Figure 3 C) matching that of the unmodified analogue (X=**T**, Y=**C**). These results demonstrate unambiguously the role played by the boronic acid and diol partners to either reactivate the DNAzyme or, if needed, suppress its activity.

## Discussion

Split DNAzymes carrying a cytosine ribonucleotide at the 3′ terminus, and a boronic acid derivative (**T^Bn^**, **Ph^Bn^** or **Napht^Bn^**) at the 5′ terminus at the split site were investigated. The objective of evaluating **Ph^Bn^** and **Napht^Bn^** in addition to **T^Bn^** was twofold. Firstly, we sought to take advantage of the greater stability of aromatic boronic acids and of the resulting boronate esters. Secondly, by positioning **Ph^Bn^** and **Napht^Bn^** in place of the last residue or after the T_12_, T_16_, T_25_, T_27_, and T_29_ positions, we wanted to evaluate the influence of flexibility and stacking interactions. It is notable that these parameters seem to have had an influence only in the case of **Dz6**, in which stacking interactions most likely participate in stabilization of the system. We have already demonstrated in the past by semiempirical calculations^[16b]^ that the electrostatic potential, sugar puckering, and C4′–oxygen distance of **T^Bn^** are almost identical to those of its natural counterpart. The present results confirm the importance of this bioisostere for efficient control of DNAzyme activity.

**Figure 4:**
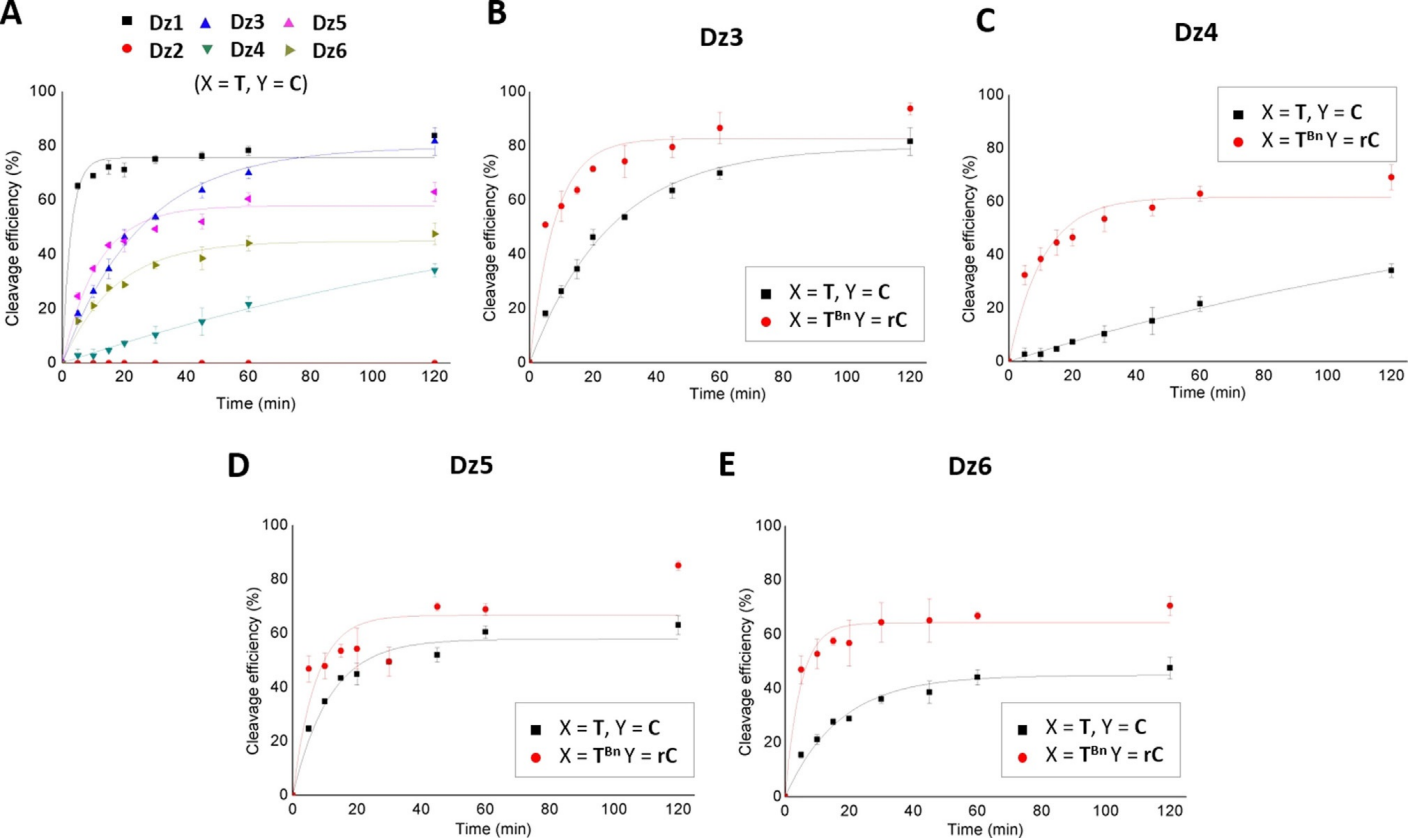
Time courses of cleavage reactions under single‐turnover conditions analyzed by a LICOR DNA sequencer. A) Parent DNAzyme **Dz1** and unmodified split DNAzymes **Dz2**–**Dz6**. DNAzyme activities of unmodified and modified split DNAzymes B) **Dz3**, C) **Dz4**, D) **Dz5**, and E) **Dz6**.

There is quite some evidence in the literature that catalysis by the 10–23 DNAzyme can be suitably described by the Michaelis–Menten model. However, most studies have not been carried out under saturating substrate concentration, but under single‐turnover conditions with the DNAzyme in excess over substrate. Normally, a simple first‐order reaction is obtained then. Nevertheless, as observed for other ribozymes, the DNAzyme cleavage reaction can follow biphasic kinetics. For an ideal DNAzyme under saturating conditions, every substrate molecule is expected to bind to a DNAzyme, and the observed rate of cleavage *k* should be equal to the sum of the forward and reverse rate constants. Biphasic kinetics may arise when an alternative conformation of the DNAzyme–substrate complex forms off the cleavage pathway and is in slow exchange with the active conformation. In this case, one would observe an initial fast rate that corresponds to the normal cleavage rate, followed by a slow rate after a certain percentage of the substrate is cleaved. Some of the curves shown in Figure 4 indeed imply that the kinetics may be more complicated than simple first‐order reactions. It is possible that with the split DNAzymes, part of the RNA substrate is trapped in alternative cleavage‐inactive conformations. If these transform into an active conformation, cleavage would be slower or follow biphasic kinetics, but the reaction eventually would reach completion. Therefore, we preferentially rely on the final percentage of cleavage for comparing the different systems. In some cases (Figure 4), we observed only a small fraction of cleaved substrate. This may indicate 1) chemically impure fragments constituting the split DNAzyme, 2) stable alternative conformations or aggregates of involved fragments, 3) stable inactive conformations of DNAzyme–substrate complex, or 4) an inactive conformation of the DNAzyme that stably binds the substrate. For DNAzymes investigated herein, it appears reasonable that, depending on the split site and the functionality at the 5′ terminus of the 3′‐DNAzyme fragment, part of the RNA substrate may bind to either the 5′ or the 3′ fragment of the DNAzyme without being cleaved, because the catalytically competent complex is not formed. Taken together, the reached percentage of cleavage appears to be a suitable measure for DNAzyme performance and functionality of the individual split DNAzymes.

Important insights have emerged from these results. The different unmodified split systems show various degrees of cleavage activity (Figure 4 A). This was surprising, since one may expect that, if split into fragments, the catalytically competent conformation is difficult if not impossible to adopt. This would apply in particular to variants **Dz2**, **Dz3**, and **Dz4**, in which the split is located in the catalytic loop (Figure 2 C). Yet, activity was observed for the unmodified split variants of **Dz3** and **Dz4** (Table 1, Figure 4). Only **Dz2** showed high sensitivity when split into fragments. In that case, no activity was observed, neither for the unmodified split variant, nor for the modified variants with potential for boronate formation (Table 1, Figure 4 A). Taken together, we have demonstrated that activity of split DNAzymes can be significantly restored by boronate ester formation, which does not require any kind of additional chemical or enzymatic activation. This is a clear advantage over other split systems that, in order to become functional, rely on chemical or enzymatic ligation of the assembled fragments.^[15]^ While the potential of **Ph^Bn^** and **Napht^Bn^** remains limited, **T^Bn^** stands out as an efficient bioisosteric analogue of natural thymidine, even when located in non‐Watson–Crick base‐paired regions, as demonstrated with **Dz3** (Figure 4 B–E). Thus, while many studies have been devoted to the analysis of functional sequence requirements in the catalytic core of the 10–23 DNAzyme,^[13]^ we here confirm and extend previous results for both 10–23[^17c^, ^20^] and 8–17 DNAzymes^[21]^ showing that, even if perturbed, unmodified split systems are able to maintain some level of functionality.

Even though the 10–23 DNAzyme is the most extensively studied representative of the DNAzyme family, structural data are rare, and the exact cleavage mechanism is still rather poorly understood. Nevertheless, a large number of biochemical studies have allowed conclusions to be drawn on the importance and functional role of nucleotides in the catalytic loop. According to the numbering used in our study and shown in Figure 2 A, G_9_ to G_14_, as well as C_21_ and G_22_ are absolutely essential, since modification at those positions greatly affects cleavage activity. Modification of C_15_ to A_20_ and A_23_ was found to only slightly affect the cleavage rate,^[13b]^ whereby T_16_ appears to be completely dispensable: it can be replaced with a C3 spacer or an abasic residue, or completely deleted without loss of activity.^[13b]^ Deletion of T_16_ was even found to increase cleavage activity.[^13a^, ^13b^, ^20b^] Furthermore, it was shown previously that a certain degree of conformational flexibility is crucial for the 10–23 DNAzyme activity. Substitution of T_16_ by an LNA analogue resulted in strong reduction of activity.^[22]^ Thus, not surprisingly, splitting the DNAzyme between C_15_ and T_16_ (as in **Dz3**) did not significantly affect activity, and even the unmodified split system cleaved up to 82 % of the substrate (Table 1).

On the contrary, T_12_ has been described being absolutely essential for activity. Structural prearrangement of some of the loop nucleotides is important for correct function, and, in particular, proper positioning of the nucleobase of T_12_ as well as of the exocyclic amino group of C_11_ seems to be required.[^13b^, ^20b^] In addition, the nonbridging oxygen atoms of the phosphate group between T_12_ and A_13_ were suggested to be involved in metal‐ion coordination.[^13b^, ^13c^, ^20b^] Substitution of loop nucleotides by a C3 spacer or an abasic site enhances flexibility of the DNAzyme loop, which has been shown to be tolerated at some positions, but not T_12_.[^13b^, ^13c^, ^20b^] Therefore, splitting the DNAzyme between C_11_ and T_12_ (as in **Dz2**) heavily affects activity, very likely due to the more flexible structure and the therefore missing required local arrangement at this site. Activity could not even be restored by the borono‐functionalized fragments, and this implies that the boronate linkage may not even have formed, as a consequence of the flexible structural environment.

The neighboring nucleotides of the scissile purine nucleotide in the RNA substrate play an important role in determining a flexible equilibrium of base pairing with the DNAzyme, thereby acting as a hinge between the catalytic loop and the RNA‐DNA helical arms.[^14b^, ^17b^]. In our DNAzyme variant, U+1 would be in equilibrium of base pairing with A_8_ or G_9_, and G−1 would be in equilibrium of base pairing with A_23_ or C_24_. These equilibria allow the catalytic loop to be properly positioned relative to the double‐helical arms and adopt the catalytically competent conformation. The results obtained for **Dz4** with the split site between C_24_ and T_25_ (Figure 2) are in agreement with this important functional role of C_24_. Activity of the unmodified split system is strongly abolished, and also formation of a boronate linkage can only partially restore it. Lastly, results obtained for **Dz5** and **Dz6**, both bearing the split site in the helical arms, mirror increased stability of the DNAzyme–substrate complex, due to formation of the boronate linkage as compared with the unmodified fragments, and as a consequence higher cleavage activity.

## Conclusion

We have reported a new concept for the activity control of split DNAzymes, through the use of two fragments, one of which is modified with a boronic acid at the 5′ end and the other with a *cis*‐diol at the 3′ end. In the presence of an RNA substrate, the assembly of the two nonfunctional fragments brings the two moieties into close proximity, and a boronate internucleoside linkage is formed spontaneously. This leads to stabilization of the DNAzyme structure and restores activity for cleavage of the RNA substrate. By varying the position of the boronate linkage we were able to characterize the key elements and the optimum split site for maintaining high levels of activity. In the long term, the reversible formation of boronate esters and the tunable boronic ester–boronate equilibrium may be a promising concept to control functional nucleic acids for the development of valuable biosensing platforms.

## Conflict of interest

The authors declare no conflict of interest.

## Supporting information

As a service to our authors and readers, this journal provides supporting information supplied by the authors. Such materials are peer reviewed and may be re‐organized for online delivery, but are not copy‐edited or typeset. Technical support issues arising from supporting information (other than missing files) should be addressed to the authors.

Supplementary
