# Supplementary material for: Boronic Acid‐Mediated Activity Control of Split 10–23 DNAzymes
Source: Chemistry. 2020 Dec 15;27(3):1138–44. doi: 10.1002/chem.202004227 (PMC7839725; doi:10.1002/chem.202004227)
Supplement: Supplementary file 1 — Supplementary [file CHEM-27-1138-s001.pdf]

Chemistry–A European Journal

Supporting Information

Boronic Acid-Mediated Activity Control of Split 10–23 DNAzymes

Mégane Debiais,[a] Amandine Lelievre,[b] Jean-Jacques Vasseur,[a] Sabine Müller,*[b] and Michael Smietana*[a]

TABLE OF CONTENTS

1. **Organic synthesis** 3
2. **NMR spectra** 7
3. **Oligonucleotide synthesis** 16
4. **Cleavage reaction** 18
5. **Gel electrophoresis analysis and time conversion curves** 19

Table 1: Conditions used for the oligonucleotide synthesis 16
Table 2: Isolated yields obtained after purification by semi-preparative RP-HPLC. 17
Table 3: *m/z* obtained by MALDI-TOF analysis for pure ONs. 18

Figure S1. ^{1}H NMR spectra of **1** 7
Figure S2. ^{13}C NMR spectra of **1** 7
Figure S3. ^{11}B NMR spectra of **1**. 8
Figure S4. ^{1}H NMR spectra of **2** 8
Figure S5. ^{13}C NMR spectra of **2**. 9
Figure S6. ^{11}B NMR spectra of **2**. 9
Figure S7. ^{31}P NMR spectra of **2**. 10
Figure S8: ^{1}H NMR spectra of **3**. 10
Figure S9. ^{13}C NMR spectra of **3**. 11
Figure S10. ^{11}B NMR spectra of **3**. 11
Figure S11. ^{31}P NMR spectra of **3**. 12
Figure S12. ^{1}H NMR spectra of **4** 12
Figure S13. ^{13}C NMR spectra of **4**. 13
Figure S14. ^{11}B NMR spectra of **4**. 13
Figure S15. ^{1}H NMR spectra of **5** 14
Figure S16. ^{13}C NMR spectra of **5**. 14
Figure S17. ^{11}B NMR spectra of **5**. 15
Figure S18. ^{31}P NMR spectra of **5**. 15
Figure S19. Gel electrophoresis analysis and time conversion curve of **Dz1** (**A**) in presence of 20 mM $MgCl_2$ and (**B**) in absence of $MgCl_2$ .... **Erreur ! Signet non défini.**
Figure S20. Gel electrophoresis analysis of **Dz2** : (**A**) X=C, Y=T and (**B**) X= **T^{Bn}**, Y= rC 19
Figure S21. Gel electrophoresis analysis and time conversion curve of **Dz3** (X=T, Y=C). 20
Figure S22. Gel electrophoresis analysis and time conversion curve of **Dz3** (X=**T^{bn}**, Y=rC). 20
Figure S23. Gel electrophoresis analysis and time conversion curve of **Dz3** (X=**T^{bn}**, Y=C) 20
Figure S24. Gel electrophoresis analysis and time conversion curve of **Dz3** (X=**Napht^{bn}**, Y=rC). 21
Figure S25. Gel electrophoresis analysis and time conversion curve of **Dz3** (X=T**Napht^{bn}**, Y=rC). 21
Figure S26. Gel electrophoresis analysis and time conversion curve of **Dz4** (X=T, Y=C) 21
Figure S27. Gel electrophoresis analysis and time conversion curve of **Dz4** (X=**T^{bn}**, Y=rC) 22
Figure S28. Gel electrophoresis analysis and time conversion curve of **Dz4** (X=**Ph^{bn}**, Y=rC). 22
Figure S29. Gel electrophoresis analysis and time conversion curve of **Dz4** (X=**Napht^{bn}**, Y=rC) 22
Figure S30. Gel electrophoresis analysis and time conversion curve of **Dz4** (X=T**Ph^{bn}**, Y=rC) 23
Figure S31. Gel electrophoresis analysis and time conversion curve of **Dz4** (X=T**Napht^{bn}**, Y=rC) 23
Figure S32. Gel electrophoresis analysis and time conversion curve of **Dz5** (X=T, Y=rC) 23
Figure S33. Gel electrophoresis analysis and time conversion curve of **Dz5** (X=**T^{bn}**, Y=rC) 24
Figure S34. Gel electrophoresis analysis and time conversion curve of **Dz5** (X=**Ph^{bn}**, Y=rC). 24
Figure S35. Gel electrophoresis analysis and time conversion curve of **Dz5** (X=**Napht^{bn}**, Y=rC) 24
Figure S36. Gel electrophoresis analysis and time conversion curve of **Dz5** (X=T**Ph^{bn}**, Y=rC) 25
Figure S37. Gel electrophoresis analysis and time conversion curve of **Dz5** (X=T**Napht^{bn}**, Y=rC) 25
Figure S38. Gel electrophoresis analysis and time conversion curve of **Dz6** (X=T, Y=rC) 25
Figure S39. Gel electrophoresis analysis and time conversion curve of **Dz6** (X=**T^{bn}**, Y=rC) 26
Figure S40. Gel electrophoresis analysis and time conversion curve of **Dz6** (X=**Ph^{bn}**, Y=rC) 26
Figure S41. Gel electrophoresis analysis and time conversion curve of **Dz6** (X=**Napht^{bn}**, Y=rC) 26
Figure S42. Gel electrophoresis analysis and time conversion curve of **Dz6** (X=T**Ph^{bn}**, Y=rC). 27
Figure S43. Gel electrophoresis analysis and time conversion curve of **Dz6** (X=T**Napht^{bn}**, Y=rC) 27
Figure S44: Gel electrophoresis analysis and time conversion curve of **Dz1** at pH 5.5 27
Figure S45: Gel electrophoresis analysis and time conversion curve of **Dz1** at pH 9.6 28

Figure S46: Gel electrophoresis analysis of **Dz4** (X=T, Y=C) at pH 5.5. 28
Figure S47: Gel electrophoresis analysis and time conversion curve of **Dz4** (X=T, Y=C) at pH 9.6..................... 28

1. Organic synthesis

Commercially available reagents were used as received. Pyridine was distilled on calcium hybrid. The reaction mixtures were monitored by TLC using silica gel plates (Merck) with fluorescent indicator (λ = 254 nm). The purification of the products was performed by flash column chromatography using silica gel 60 (43–63 µm) from Merck-Millipore. NMR analyses were made on a Bruker spectrometer 400 MHz at 20 °C. Chemical shifts are reported in parts per million (ppm) and are referenced to the residual solvent resonance as the internal standard (CD_3CN δ = 1.94 for ^1H NMR and CD_3CN δ = 1.32 for ^{13}C NMR). Data are reported as follows: chemical shift, multiplicity (standard abbreviations), coupling constant (Hz), and integration. HRMS were recorded on a Micromass Q-TOF spectrometer with an electrospray ionisation (ESI) in negative or positive mode. All the oligonucleotide syntheses were conducted on an ABI 394 DNA/RNA synthesizer by classical phosphoramidite chemistry with commercial phosphoramidites and CPG (Control Pore Glass) solid support using standard conditions. Crude sequences were purified by semi-preparative HPLC Dionex 600 in reverse phase with a gradient of buffer B (TEAAc 0.05M + 80% ACN) in buffer A (TEAAc 0.05M + 1% ACN) at 30 °C at a flow rate of 4 mL/min. Purified DNA samples were analysed by analytic HPLC with a gradient of buffer B (TEAAc 0.05M + 80% ACN) in buffer A (TEAAc 0.05M + 1% ACN) at a flow of 1 mL/min. MALDI-TOF mass spectra were recorded on an Axima assurance spectrometer (Shimadzu Biotech) using 1 µL of purified DNA sample mixed with 5 µL of a 6-aza-2-thiothimine (ATT) saturated matrix and ammonium citrate (0.1 M) as co-matrix. Samples were then spotted on a stainless steel plate and air dried before analysis.

Synthesis of the compound 1-(5'-deoxy thymidin-5'-yl)methylboronic acid has already been described by Luvino *et al.*[1] and Gimenez Molina *et al.*[2]

1-(5'-deoxy-5'-methylthymidin-6'-yl)-1,3,6,2-dioxazaborocane-4,8-dione (1)

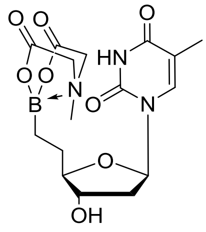

$C_{16}H_{22}BN_3O_8$
M=395.18 g/mol

An oven-dried flask topped with a Dean-Stark apparatus and condenser was charged sequentially with 1-(5'-deoxy thymidin-5'-yl)methylboronic acid (315.0 mg, 1.11 mmol),[1] MIDA (232.0 mg, 2.22 mmol), anhydrous benzene (3.6 mL) and DMSO (0.4 mL). The Dean-Stark trap was filled with benzene and the resulting mixture was heated at reflux for 20 hours (reaction monitored by TLC). The solution was cooled to room temperature, concentrated under reduced pressure and the resulting residue was purified by flash column chromatography over silica gel (EA/Acetone, 95:5) to provide compound **1** (270.0 mg, 62 %). R_f **:** 0.10 (EA/Acetone, 95/5).

^{1}H NMR (400MHz, CD_3CN) δ (ppm) : 0.58-0.80 (m, 2H), 1.56-1.72 (m, 2H), 1.84 (s, 3H), 2.09-2.22 (m, 2H), 2.86 (s, 3H), 3.35 (bs, 1H), 3.69-3.74 (m, 1H), 3.80 (d, J = 17.2 Hz, 2H), 3.94 (d, J = 16.8 Hz, 2H), 4.09-4.15 (m, 1H), 6.16 (t, J = 6.8 Hz, 1H), 7.25 (s, 1H), 8.96 (bs, 1H).

^{13}C NMR (100MHz, CD_3CN) δ (ppm) : 12.6, 29.1, 40.0, 46.7, 62.8, 74.5, 84.9, 89.5, 111.4, 136.7, 151.7, 164.9, 169.3.

^{11}B NMR (128MHz, CD_3CN) δ (ppm) : 13.29.

HRMS (ESI$^+$) : m/z calculated for $C_{16}H_{23}BN_3O_8$ $[M+H]^+$: 396.1578, found : 396.1581.

O-(2-cyanoethyl)- N,N-diisopropyl-O-[1-(5'-deoxy-5'-methylthimidin-6'-yl)-1,3,6,2-dioxazaborocane-4,8-dione] phosphoramidite (2)

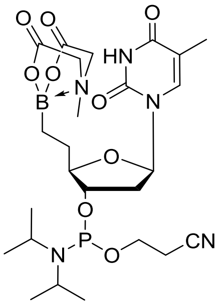

$C_{25}H_{39}BN_5O_9P$
M=595.40 g/mol

Compound **1** (270.0 mg, 0.68 mmol) was dissolved in anhydrous acetonitrile (5 mL) passed through alumina and the resulting solution was treated by dropwise addition of DIEA (267 µL, 1.57 mmol) and bis(diethylamino)chlorophosphine (305 µL, 1.37 mmol). The reaction mixture was stirred at room temperature for 3 hours (reaction monitored by TLC). The crude material was concentrated under reduced pressure and immediately purified by flash column chromatography over silica gel (EA/Acetone/Et_3N, 60:30:10) to lead to compound **2** (150.0 mg, 37 %). R_f: 0.30 (EA/Acetone/Et_3N, 60:30:10).

^{1}H NMR (400MHz, CD_3CN) δ (ppm) : 0.53-0.76 (m, 2H), 1.14 (s, 6H), 1.55-1.75 (m, 2H), 1.81 (s, 3H), 2.13-2.37 (m, 2H), 2.64 (q, J = 10.4 Hz, 5.6 Hz, 2H), 2.83 (s, 3H), 3.54-3.63 (m, 2H), 3.67-3.76 (m, 2H), 3.79 (dd, J = 16.8 Hz, 4 Hz, 2H), 3.84-3.89 (m, 1H), 3.95 (dd, J = 17.2 Hz, 2.8 Hz, 2H), 4.26-4.34 (m, 1H), 6.13 (t, J = 6.4 Hz, 1H), 7.25 (s, 1H).

^{13}C NMR (100MHz, CD_3CN) δ (ppm) : 12.6, 20.9, 24.8, 24.9, 29.0, 39.0, 44.0, 46.6, 59.3, 62.8, 76.6, 85.0, 88.3, 111.5, 119.6, 136.8, 151.6, 162.1, 169.4.

^{11}B NMR (128MHz, CD_3CN) δ (ppm) : 13.57.

^{31}P NMR (162MHz, CD_3CN) δ (ppm) : 147.56.

HRMS (ESI$^-$) : m/z calculated for $C_{25}H_{38}BN_5O_9P$ $[M-H]^-$: 594.2500 found : 594.2499.

2-cyanoethyl (4-(4,4,5,5-tetramethyl-1,3,2-dioxaborolan-2-yl)) diisopropylphosphoramidite (3)

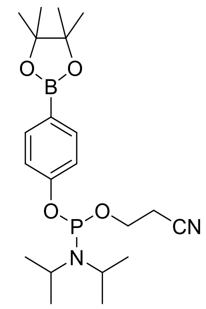

$C_{21}H_{34}BN_2O_4P$
M=420,30 g/mol

4-(4,4,5,5-tetramethyl-1,3,2-dioxaborolan-2-yl)phenol (150.0 mg, 0.68 mmol) was dissolved in anhydrous DCM (2 mL) passed through alumina and were successively dropwise added DIEA (267.0 µL, 1.57 mmol) and bis(diethylamino)chlorophosphine (304.0 µL, 1.36 mmol). The mixture was stirred at room temperature for 3 hours (reaction monitored by TLC). The crude product was evaporated under reduced pressure and purified by flash column chromatography over silica gel (Cyclohexane/EA/Et_3N, 80:10:10) to provide compound **3** (120.0 mg, 42 %) as a white solid. R_f : 0.55 (Cyclohexane/EA/Et_3N, 80:10:10)

^{1}H NMR (400MHz, $CDCl_3$) δ (ppm) : 1.11 (d, J = 6.8 Hz, 6H), 1.19 (d, J = 6.8 Hz, 6H), 1.29 (s, 12H), 2.61 (td, J = 6.4 Hz, 2 Hz, 2H), 3.64-3.73 (m, 2H), 3.86-3.92 (m, 2H), 7.00 (dd, J = 8.4 Hz, 1.6 Hz, 2H), 7.69 (d, J=8.4 Hz, 2H).

^{13}**C NMR** (100MHz, $CDCl_3$) δ (ppm) : 20.4, 24.4, 24.9, 43.7, 58.9, 83.7, 117.5, 119.2, 136.5, 157.1.

^{11}**B NMR** (128MHz, $CDCl_3$) δ (ppm) : 30.75.

^{31}**P NMR** (162MHz, $CDCl_3$) δ (ppm) : 146.19.

HRMS (ESI$^-$) : *m/z* calculated for $C_{21}H_{33}BN_2O_4P$ $[M-H]^-$: 420.2456, found : 420.2458.

2-(6-hydroxynaphtalen-2-yl)-6-methyl-1,3,6,2-dioxazaborocane-4,8-dione (4)

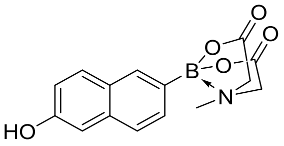

$C_{15}H_{14}BNO_5$
M=299,10 g/mol

An oven-dried flask topped with a Dean-Stark apparatus and condenser was charged sequentially with (6-hydroxynaphtalen-2-yl)boronic acid (150.0 mg, 0.80 mmol), MIDA (120.0 mg, 0.80 mmol), anhydrous benzene (9 mL) and DMSO (1 mL). The Dean-Stark trap was filled with benzene and the resulting mixture was heated at reflux for 20 hours (reaction monitored by TLC). The solution was cooled to room temperature, concentrated under reduced pressure and the resulting residue was purified by flash column chromatography over silica gel (DCM/MeOH, 90:10) to afford compound **4** (190.0 mg, 80 %) as a white solid. R_f : 0.35 (DCM/MeOH, 90:10).

^{1}**H NMR** (400MHz, CD_3CN) δ (ppm) : 2.50 (s, 3H, $CH_{3\ MIDA}$), 2.91 (s, 1H), 3.92 (d, *J* = 16.8 Hz, 2H), 4.09 (d, *J* = 16.8 Hz, 2H), 7.10 (dd, *J* = 9.2 Hz, 2.8 Hz, 1H), 7.16 (d, *J* = 2.4 Hz, 1H), 7.49 (dd, *J* = 8.4 Hz, 1.2 Hz, 1H), 7.69 (d, *J* = 8.4 Hz, 1H), 7.79 (d, *J* = 8.8 Hz, 1H), 7.93 (s, 1H).

^{13}**C NMR** (100MHz, CD_3CN) δ (ppm) : 48.5, 63.0, 109.7, 119.1, 126.6, 129.2, 130.6, 131.1, 133.8, 136.5, 156.4, 169.7.

^{11}**B NMR** (128MHz, CD_3CN) δ (ppm) : 11.89.

HMRS (ESI$^+$) : *m/z* calculated for $C_{15}H_{15}BNO_5$ $[M+H]^+$: 300.1043, found : 300.1044.

2-cyanoethyl (6-(6-methyl-4,8-dioxo-1,3,6,2-dioxazaborocan-2-yl)naphtalen-2-yl) diisopropylphosphoramidite (5)

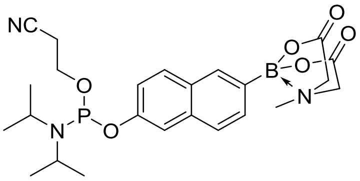

$C_{24}H_{31}BN_3O_6P$
M=499,20 g/mol

Compound **4** (190.0 mg, 0.64 mmol) was dissolved in of anhydrous acetonitrile (4 mL) passed through alumina and the resulting mixture was treated by dropwise addition of DIEA (248.0 µL, 1.46 mmol) and bis(diethylamino)chlorophosphine (283.0 µL, 1.27 mmol). The mixture was stirred at room temperature for 3 hours (reaction monitored by TLC). The solution was concentrated under reduced pressure and the crude material was purified by flash column chromatography over silica gel (DCM/Acetone/Et_3N, 70:20:10) to provide compound **5** (70.0 mg, 22 %) as a white solid. R_f : 0.50 (DCM/Acetone/Et_3N, 70:20:10).

^{1}**H NMR** (400MHz, CD_3CN) δ (ppm) : 1.21 (d, *J* = 6.8 Hz, 6H), 1.26 (d, *J* = 6.8 Hz, 6H), 2.54 (s, 3H), 2.75 (t, *J* = 6 Hz, 2H), 2.84 (s, 1H), 3.75-2.85 (m, 2H), 3.89-4.01 (m, 2H), 3.97 (d, *J* = 17.2 Hz, 2H), 4.14 (d, *J* = 17.2 Hz, 2H), 7.27 (dd, *J* = 8.8 Hz, 2.4 Hz, 1H), 7.53 (bs, 1H), 7.57 (dd, *J* = 8.4 Hz, 1.2Hz, 1H), 7.80 (d, *J* = 8.4 Hz, 1H), 7.88 (d, *J* = 8.8 Hz, 1H), 8.02 (s, 1H).

^{13}**C NMR** (100MHz, CD_3CN) δ (ppm) : 21.0, 24.8, 44.5, 48.5, 60.0, 62.9, 115.5, 119.6, 122.3, 127.2, 130.4, 130.8, 131.0, 133.8, 135.9, 153.7, 169.8.

^{11}**B NMR** (128MHz, CD_3CN) *δ* (ppm) : 11.99.

^{31}**P NMR** (162MHz, CD_3CN) *δ* (ppm) : 146.18.

HMRS (ESI$^+$) : *m/z* calculated for $C_{24}H_{32}BN_3O_6P$ $[M+H]^+$: 500.2122, found : 500.2127.

2. NMR spectra

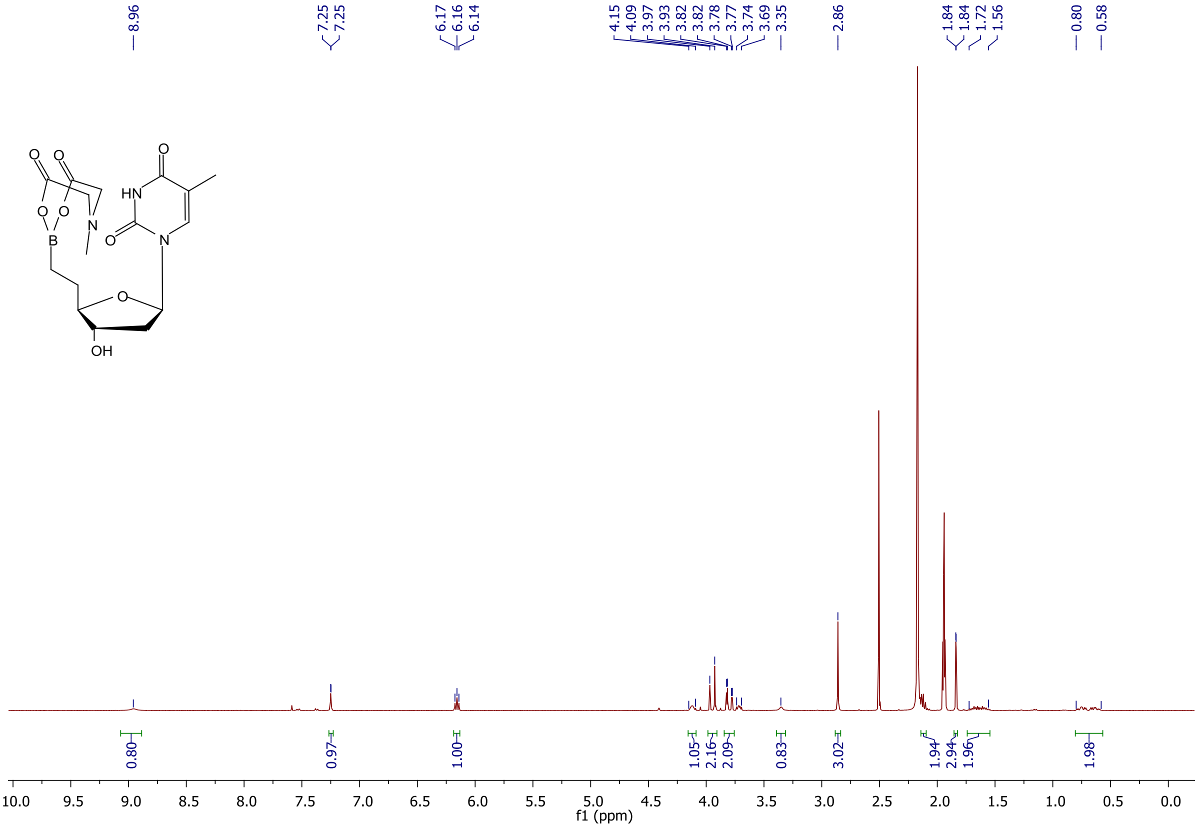

Figure S1. ^{1}H NMR spectra of **1**.

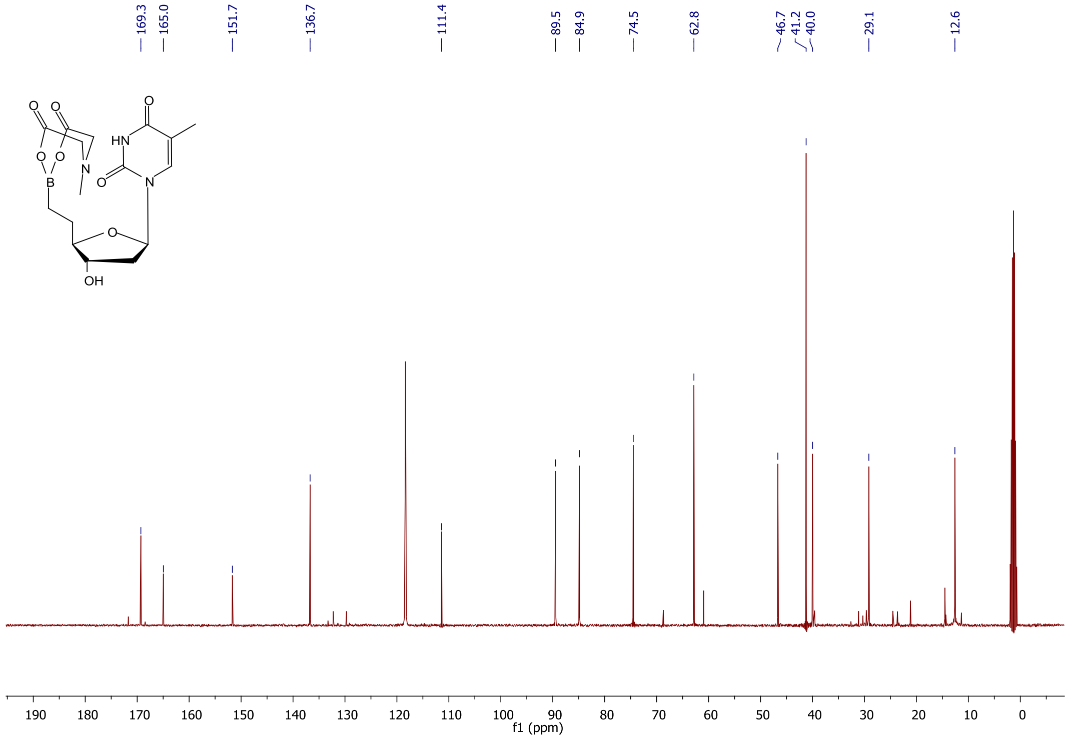

Figure S2. ^{13}C NMR spectra of **1**.

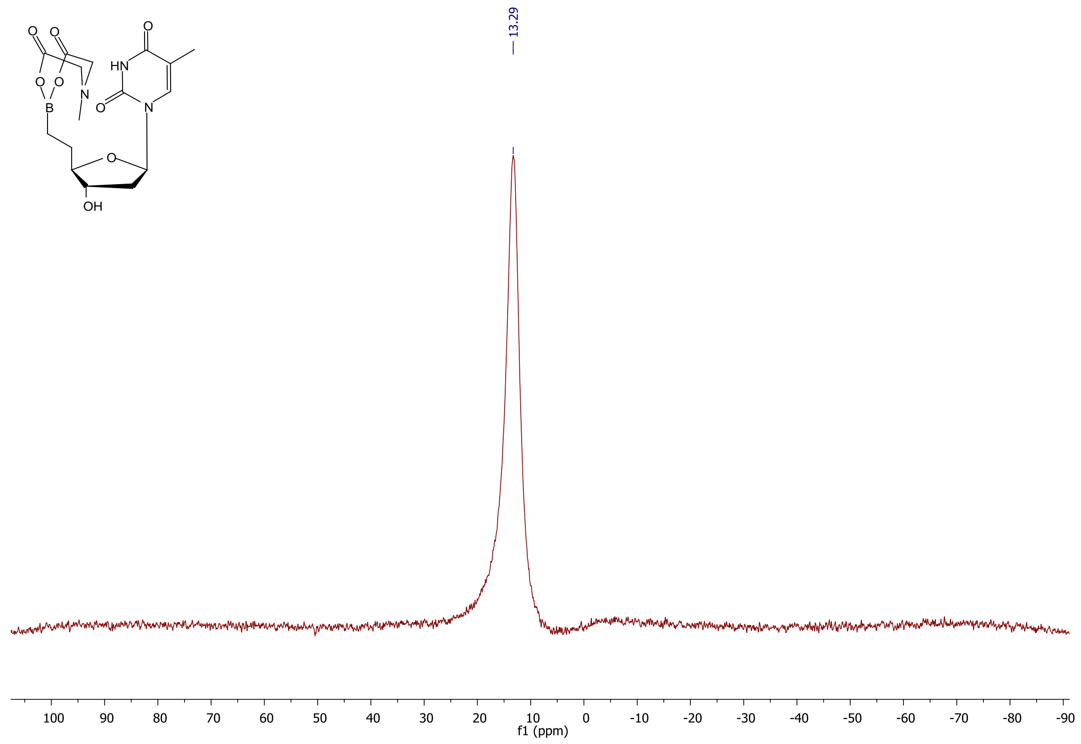

Figure S3. ^{11}B NMR spectra of **1**.

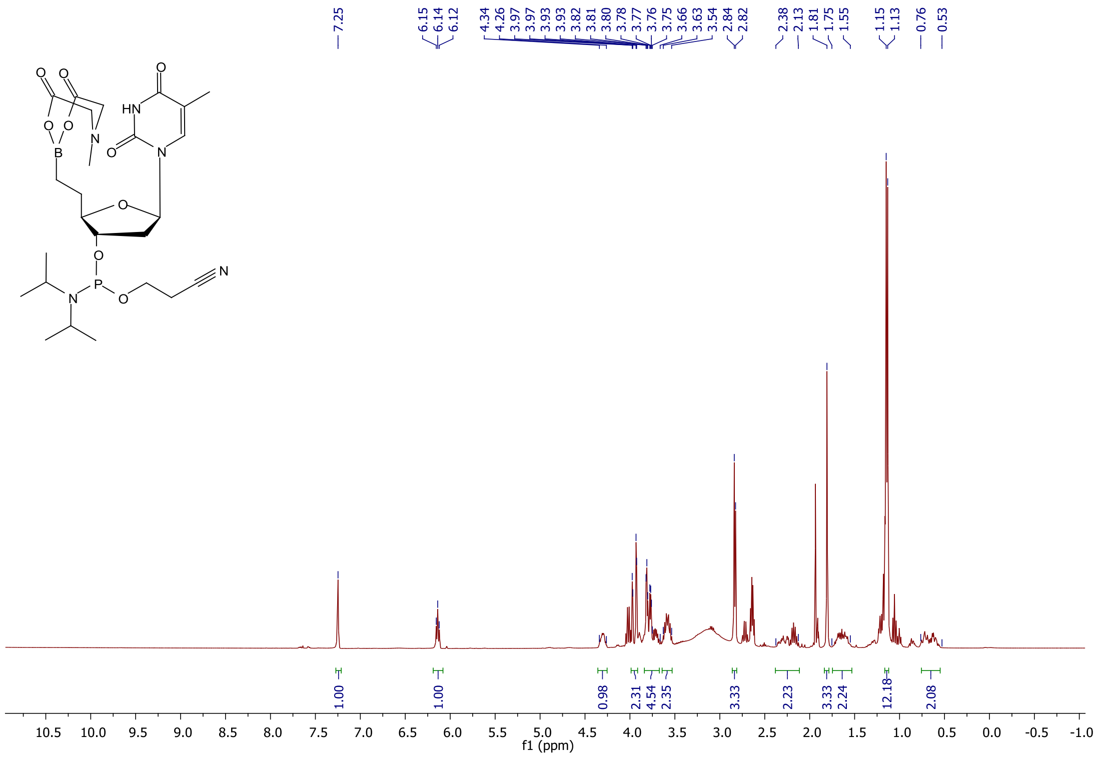

Figure S4. ^{1}H NMR spectra of **2**.

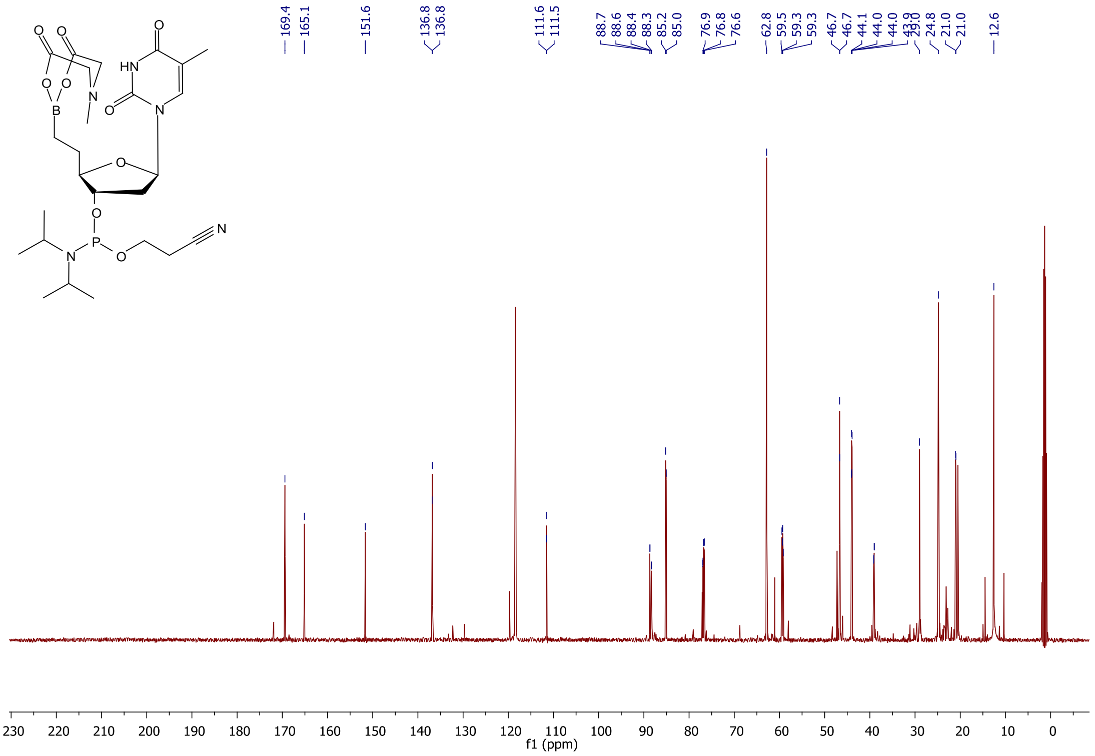

Figure S5. ^{13}C NMR spectra of **2**.

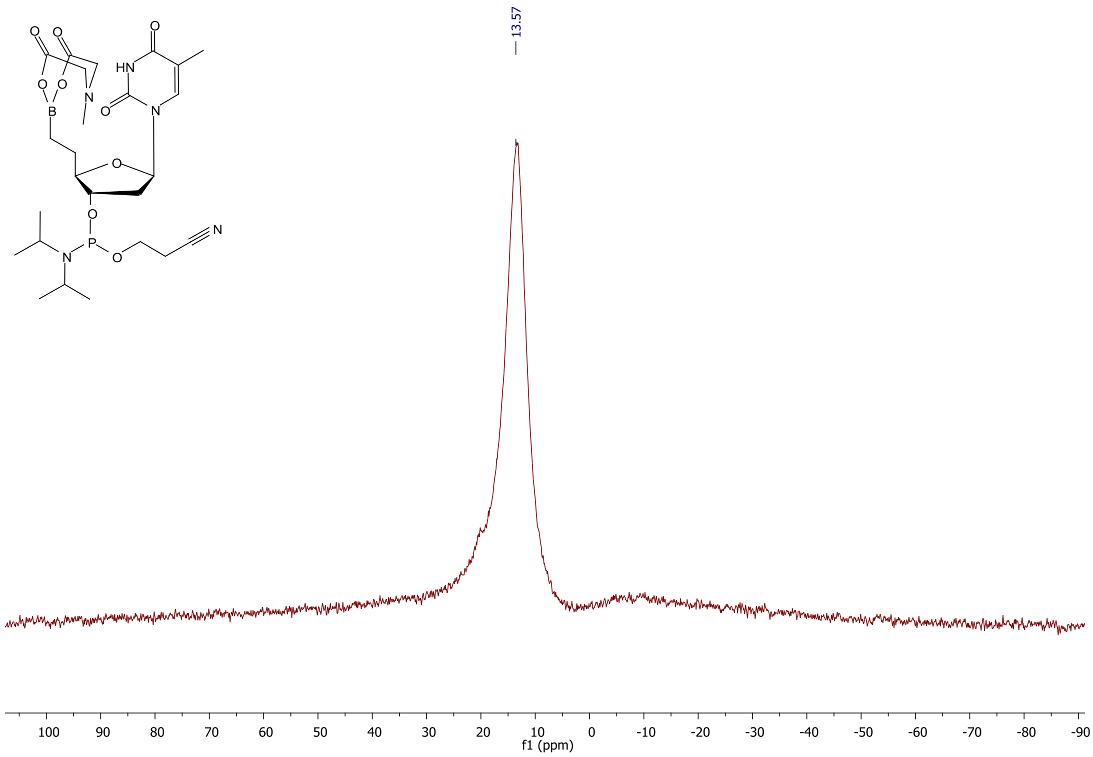

Figure S6. ^{11}B NMR spectra of **2**.

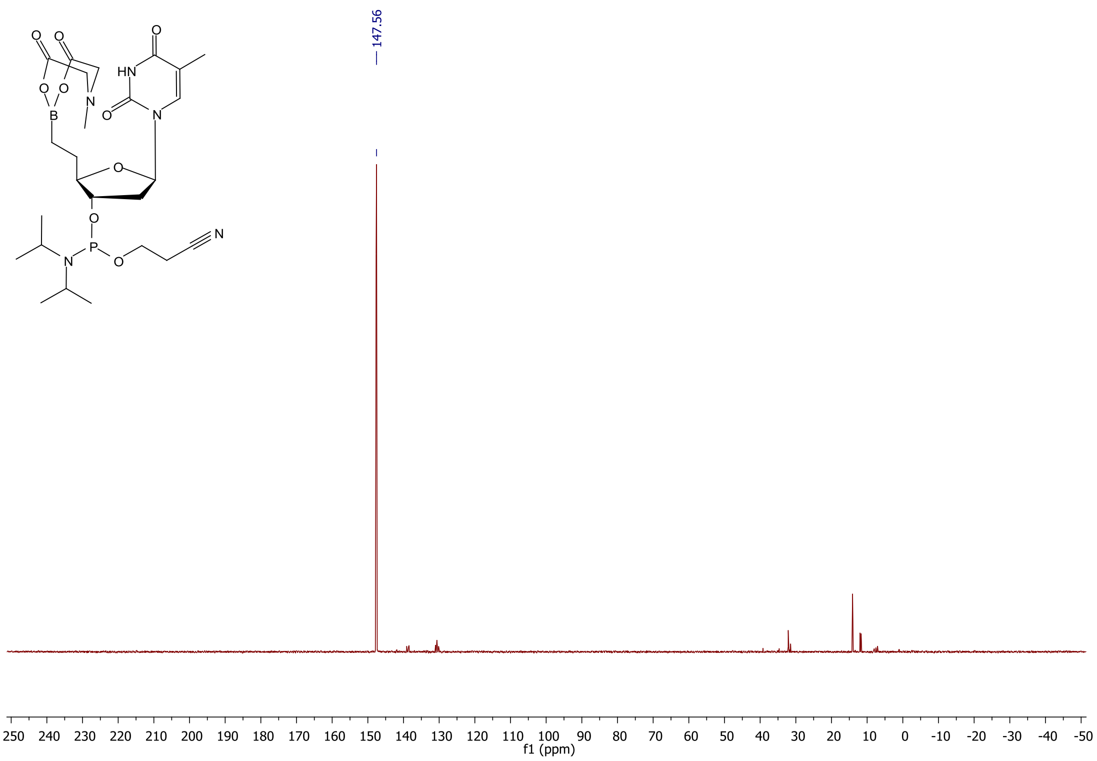

Figure S7. ^{31}P NMR spectra of **2**.

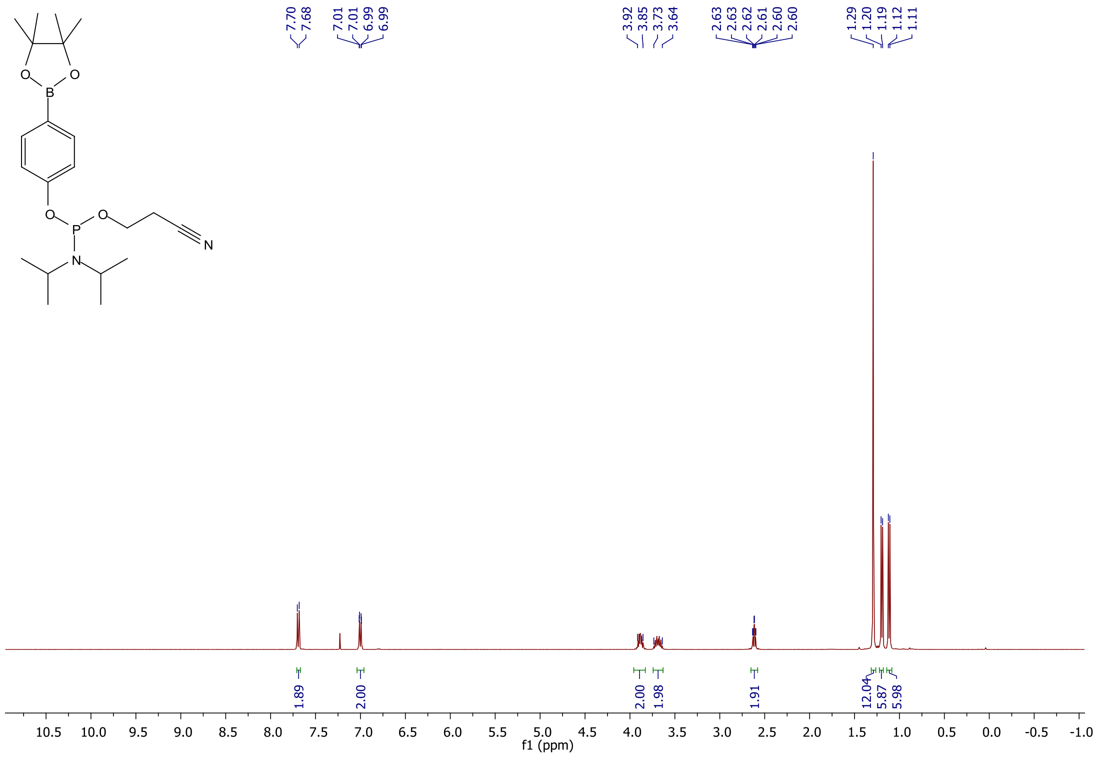

Figure S8: ^{1}H NMR spectra of **3**.

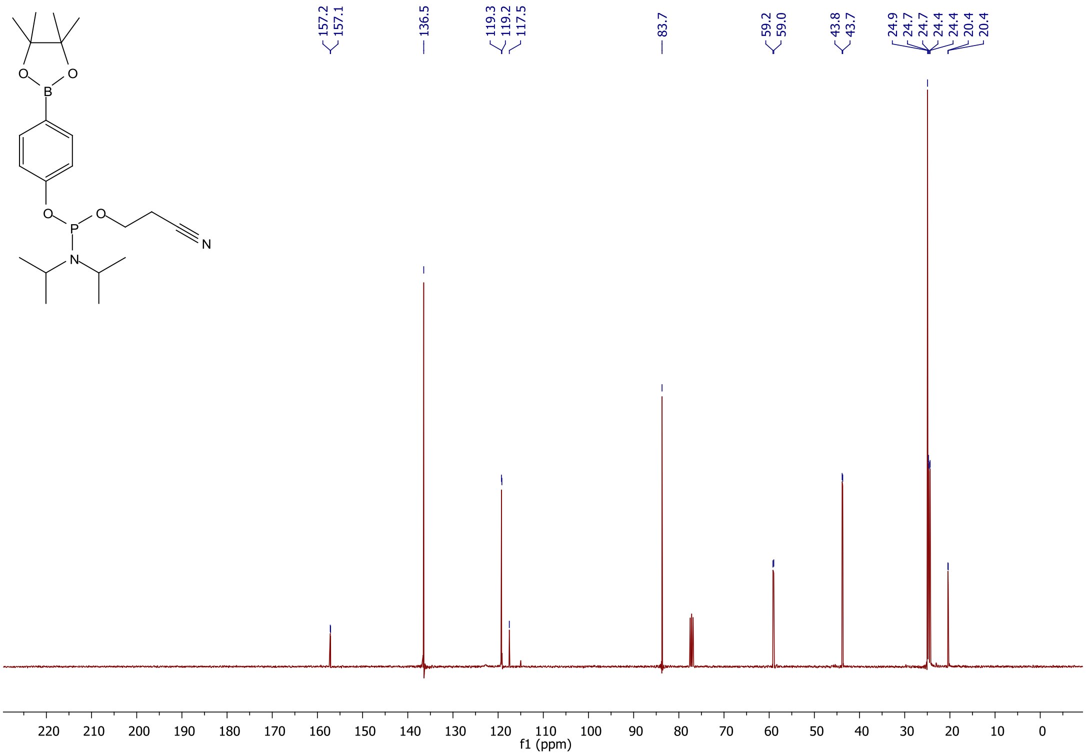

Figure S9. ^{13}C NMR spectra of **3**.

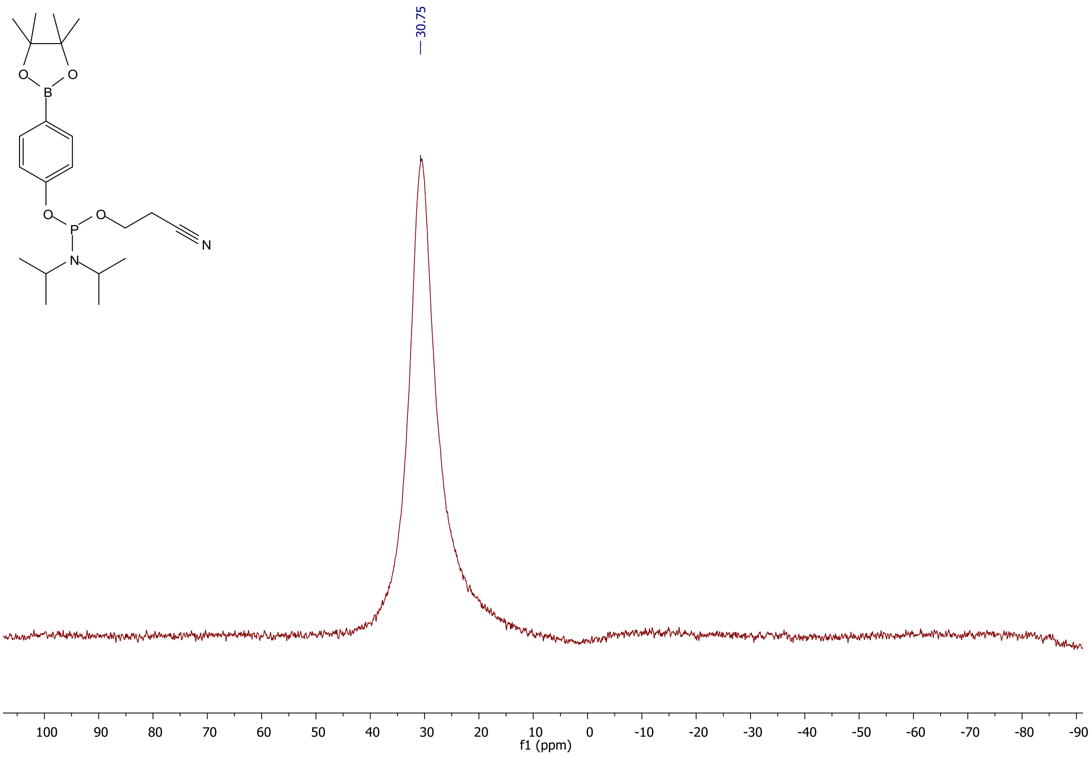

Figure S10. ^{11}B NMR spectra of **3**.

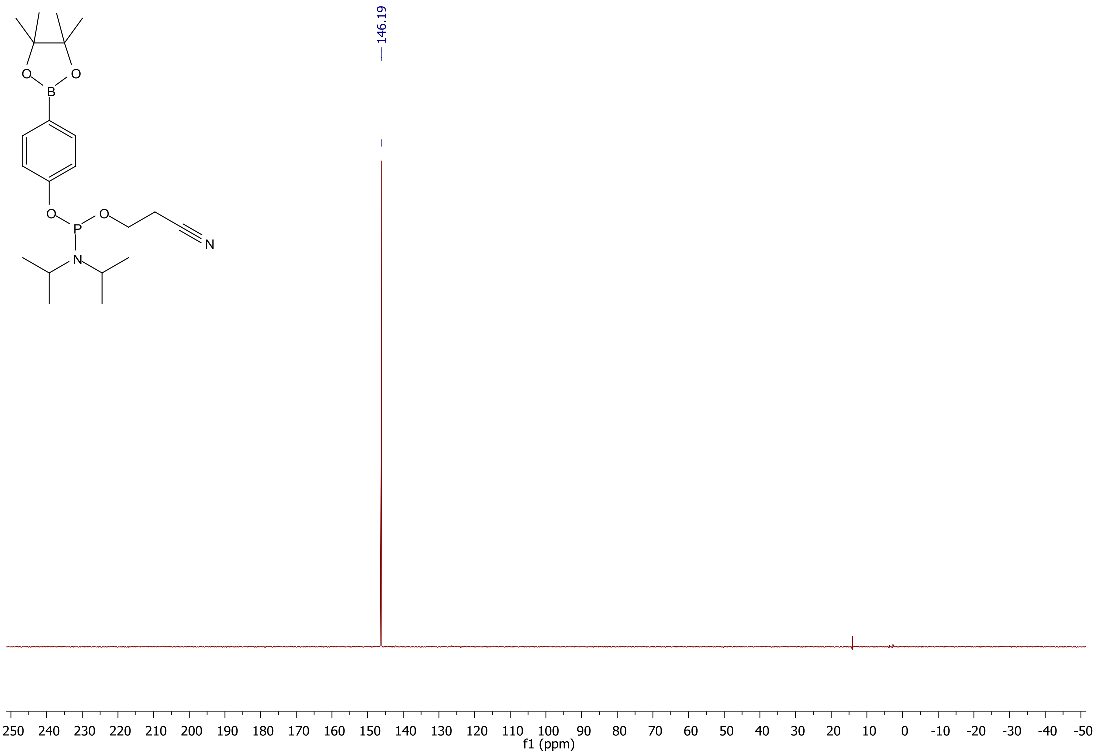

Figure S11. ^{31}P NMR spectra of **3**.

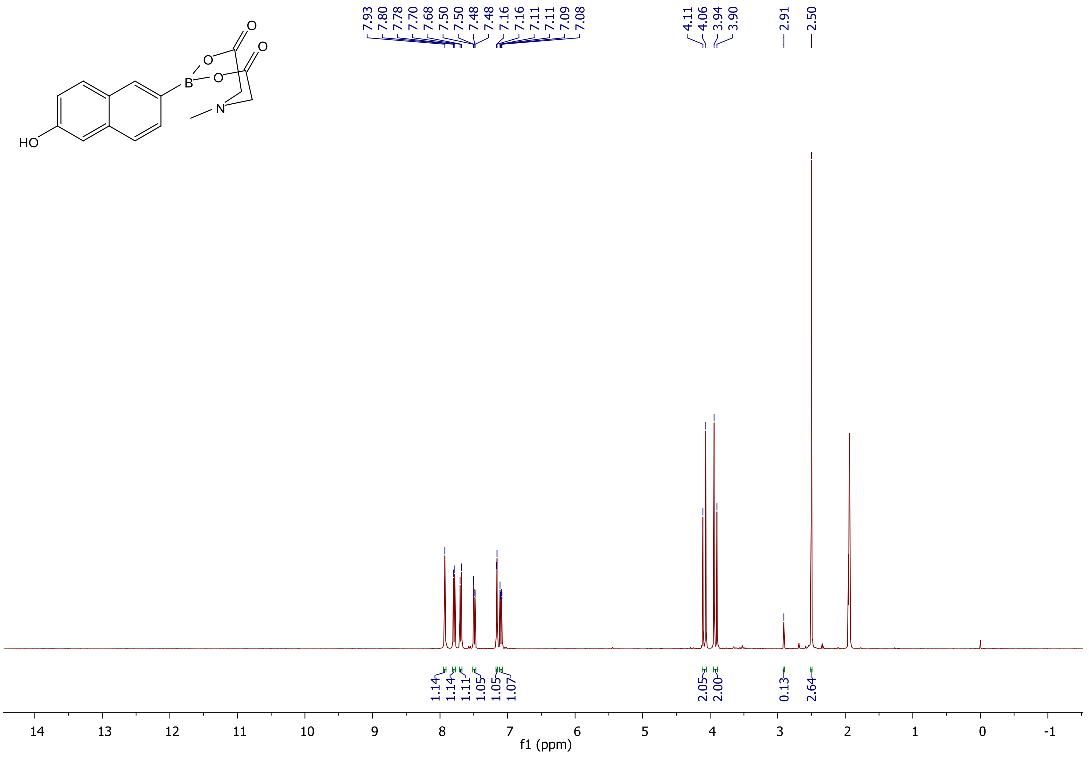

Figure S12. ^{1}H NMR spectra of **4**.

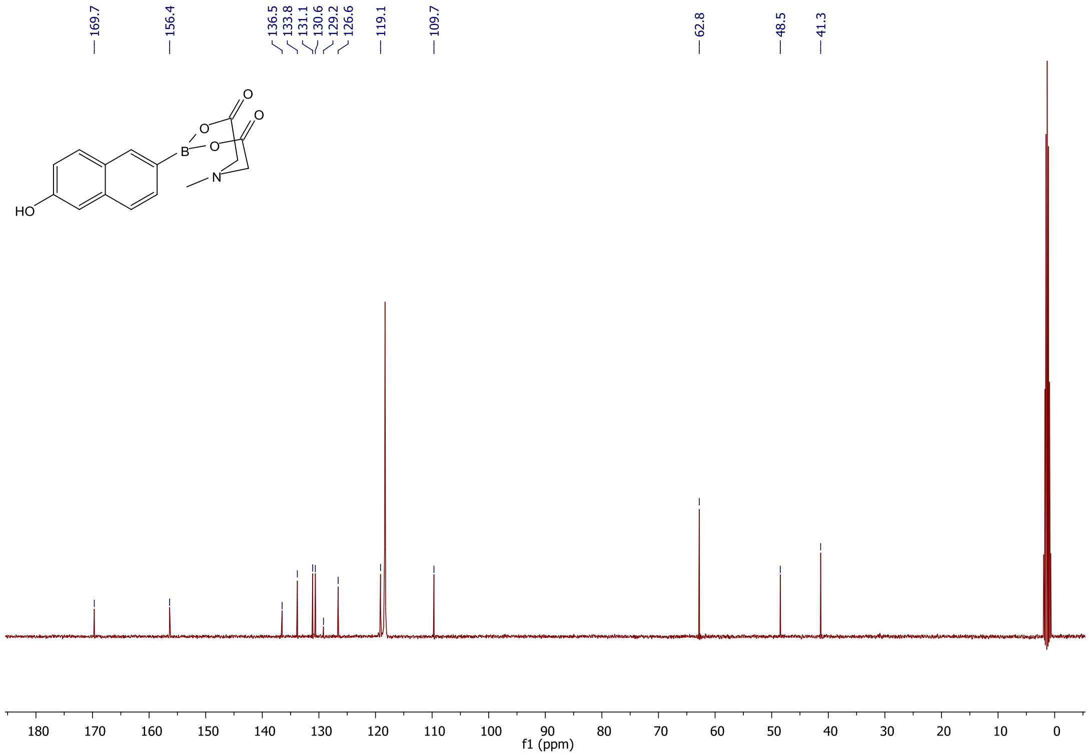

Figure S13. ^{13}C NMR spectra of **4**.

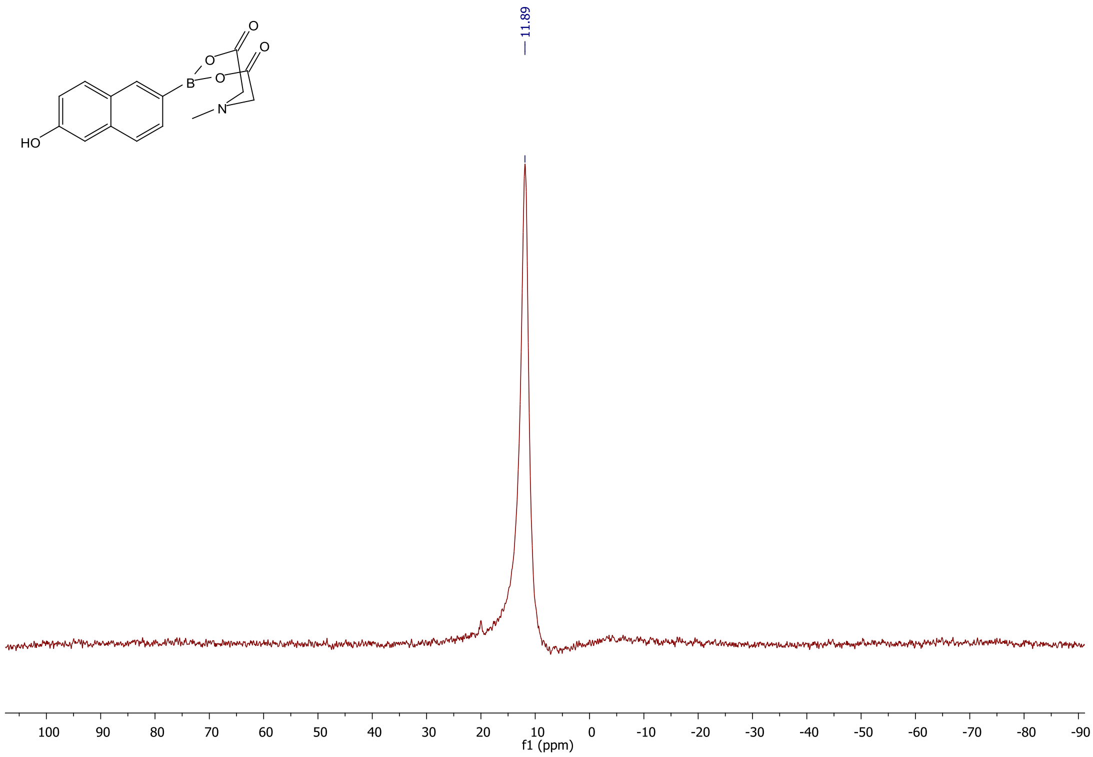

Figure S14. ^{11}B NMR spectra of **4**.

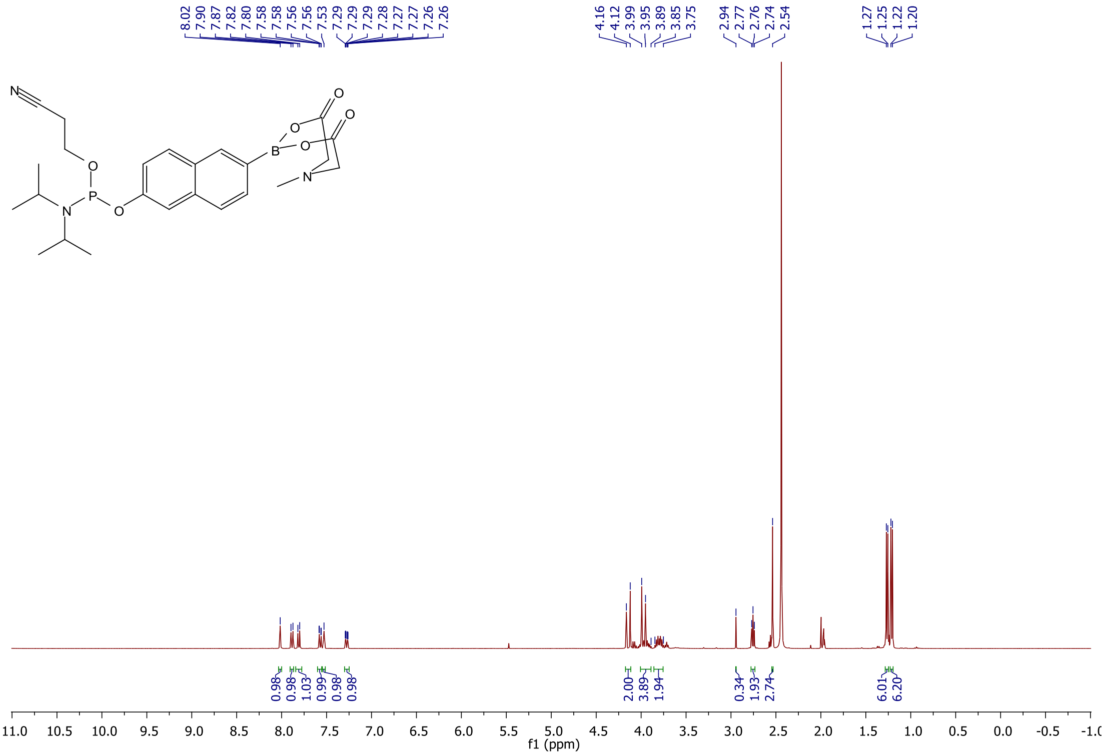

Figure S15. ^{1}H NMR spectra of **5**.

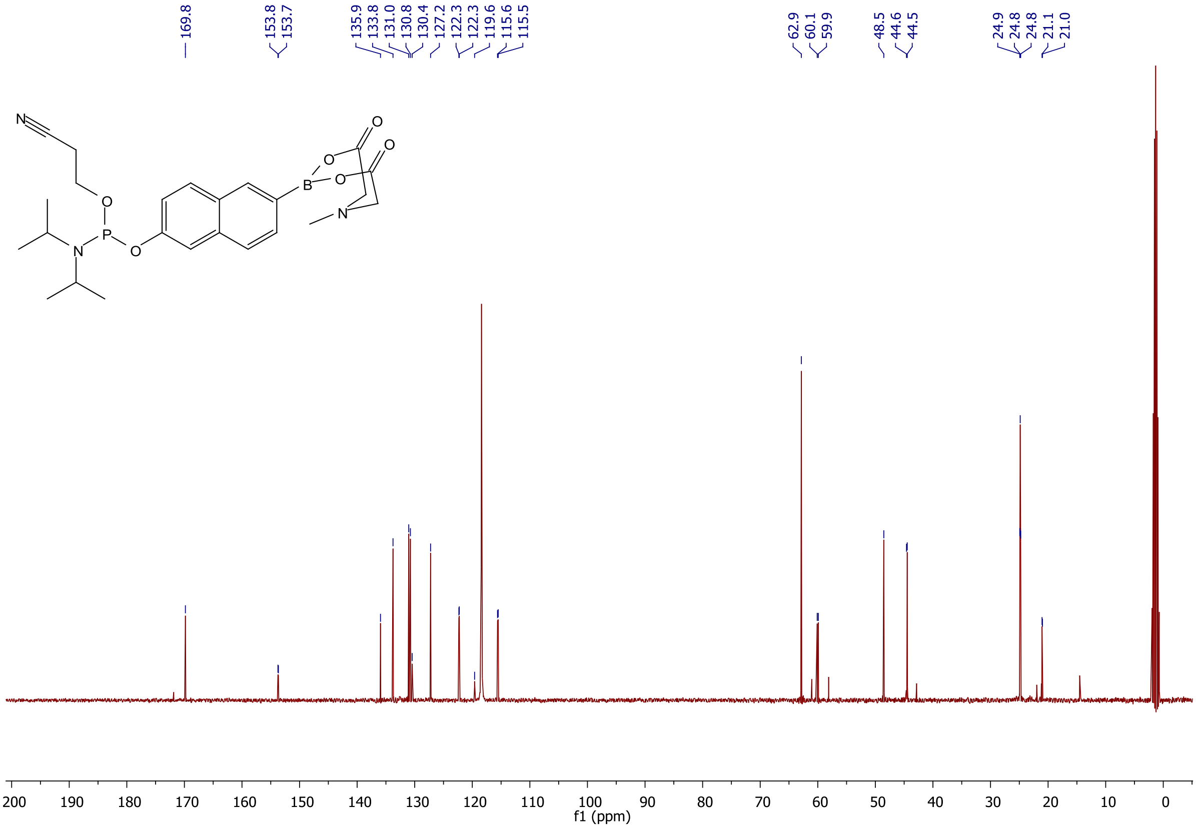

Figure S16. ^{13}C NMR spectra of **5**.

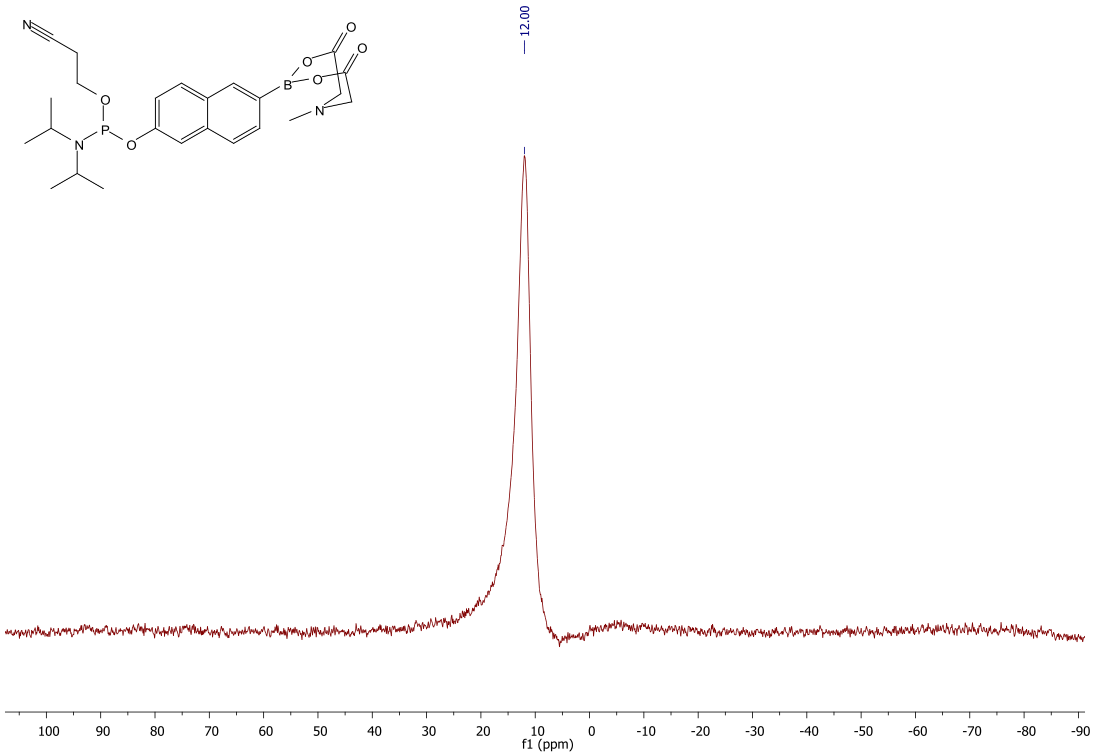

Figure S17. ^{11}B NMR spectra of **5**.

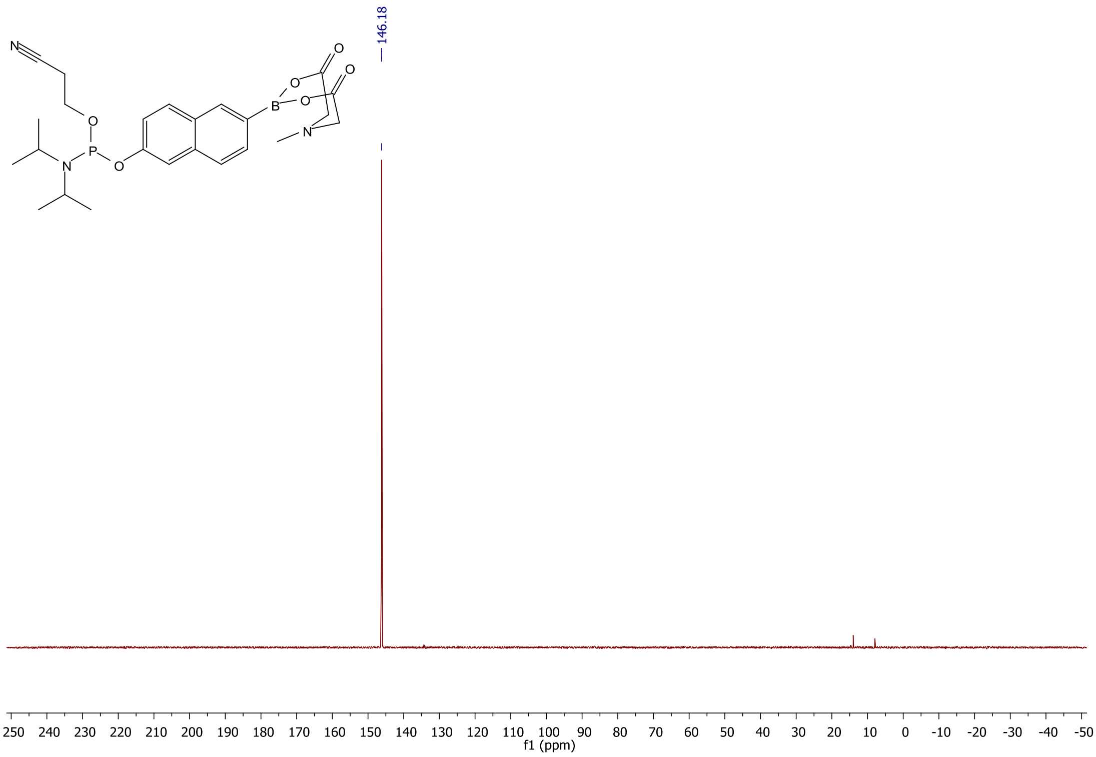

Figure S18. ^{31}P NMR spectra of **5**.

3. Oligonucleotide synthesis

All DNA syntheses were conducted on an ABI 394 DNA/RNA synthesizer by classical phosphoramidite chemistry with commercial phosphoramidites and CPG (Control Pore Glass) solid support using standards conditions. Crude sequences were purified by semi-preparative HPLC Dionex 600 in reverse phase with UV detection at 260 nm, a VP 250/10 Nucleodur C18 Htec 5 µm column, a gradient of buffer B (TEAAc 0.05M + 80% ACN) in buffer A (TEAAc 0.05M + 1% ACN) at 30°C at a flow of 4mL/min. Purified DNA samples were analysed by analytic HPLC with a EC 75/4.5 Nucleodur 100-3 C18 column, a gradient of buffer B (TEAAc 0.05M + 80% ACN) in buffer A (TEAAc 0.05M + 1% ACN) at a flow of 1mL/min. MALDI-TOF mass spectra were recorded on an Axima assurance spectrometer (Shimadzu Biotech) with a N_2 laser (337 nm) using 1µL of purified DNA sample mixed with 5µL of 6-aza-2-thiothimine (ATT) saturated matrix and ammonium citrate (0.1M) as co-matrix. Samples were then spotted on a stainless steel plate and air dried before analysis. Oligonucleotides dosage were made on a Varian Cary 300 Bio UV spectrometer with a UV detection at 260, 488 and 580 nm. DNAzymes catalytic activities were studied by gel electrophoresis using a LI-COR 4300 DNA sequencer.

Automated synthesis

Table 1: Conditions used for the oligonucleotide synthesis

Step	Reaction	Reagents	Time (s)
1	Detritylation	3% TCA/DCM	65
2	Coupling	Unmodified : 0.1M amidite/ACN + 0.3M BMT/ACN	30
		**Ph^{Bn} :** 0.1M amidite/ACN + 0.3M BMT/ACN	180
		**$Napht^{Bn}$:** 0.05M amidite/ACN + 0.3M BMT/ACN	180
		**T^{bn} :** 2x0.05M amidite/ACN + 0.3M BMT/ACN	1200
3	Capping	Ac_2O/THF/Pyridine + 10% NMI/THF	10
4	Oxidation	0.1M I_2/THF/H_2O/Pyridine	15

Deprotection, analysis and purification of oligonucleotides

DNA oligonucleotides

Deprotection and cleavage of the DNA sequences from the solid support was achieved upon mixing with concentrated NH_4OH at 55 °C for 5 h. Ammoniac was then evaporated in SpeedVac and oligonucleotides were analysed by analytic RP-HPLC and MALDI-TOF with a ATT matrix and ammonium citrate as co-matrix in negative mode. Crude oligonucleotides were then purified by semi-preparative RP-HPLC.

RNA oligonucleotides (substrate)

The RNA substrate was synthesized on a Gene Assembler using commercial phosphoramidites (2'-*O*-TBDMS protection) and 2'-*O*-propargyl Guanosine-3'-CPG. Deprotection and cleavage of the RNA sequences from the solid support was performed for 40 min at 65°C with a mixture of 32 % ammonia and 8 M methylamine in ethanol (1:1 v/v). The support was then washed twice with 150 µL of a solution of water and ethanol (1:1 v/v) and the combined liquids were lyophilized. TBDMS groups were removed by incubation with a mixture of triethylamine trifluoride and DMF (3:1 v/v) at 55°C for 1.5h. The reaction was stopped by addition of 0.25 volumes of water and the RNA was precipitated with 20 mL of n-butanol in a Falcon tube. The RNA was collected by centrifugation for 40 min at room temperature and purified by denaturing PAGE. The desired RNA was eluted from the gel with 0.3 M sodium acetate (pH=5.5) and precipitated from ethanol.

Labeling of the RNA substrate with $ATTO_{680}$ (3'-end)

The deprotected and purified oligonucleotide (6 nmol) was solved in 50 µl sodium carbonate buffer, to which 50 µg of dry $ATTO_{680}$-azide was added. A mixture of 50 µL $CuSO_4$ (1 mM in phosphate buffer 0.1 M, pH 7) and 50 µL tris(3-hydroxypropyltriazolylmethyl)amine (5 mM in phosphate buffer 0.1 M, pH 7) was saturated with argon for 5 min and added to the RNA solution, which was again degassed with argon for 5 min. 70 µL of freshly prepared sodium ascorbate solution (8 mM) was saturated with argon for 10 min and added to the reaction mixture. Finally, 240 µL phosphate buffer (0.1 µM, pH 7) were added to the solution. After incubation for 4 h at 37 °C, the RNA was precipitated from ethanol and the labelled product was isolated by RP-HPLC (EC 250/4 Nucleodur 100-5 C18, cv = 3.142 mL) with buffer A (0.1 M TEAAc, 5% acetonitrile) and buffer B (0,1 M TEAAc, 30% acetonitrile) with a four step gradient at a flow rate of 0.5 mL/min: 0% buffer B for 2 cv, 50% buffer B for 5 cv, 80% buffer B for 25 cv and 100% buffer B for 2 cv. The isolated sample was desalted by gel filtration to give 1,4 nmol of labelled RNA.

Table 2: Isolated yields obtained after purification by semi-preparative RP-HPLC.

Name	Sequence 5' → 3' [a]	Yield (nmol / (%))[b]
**Substrate**	r(GGAGAGAGAUGGGUGCG)-ATTO680[c]	1.38 (23)[d]
**Dz1**	d(CGCACCCAGGCTAGCTACAACGACTCTCTCCG)	118.6 (12)
**Dz2**	d(CGCACCCAGGC)	470.4 (47)
	d(CGCACCCAGG)rC	442.0 (44)
	d(TAGCTACAACGACTCTCTCCG)	42.01 (4)
	T^{Bn}-d(AGCTACAACGACTCTCTCCG)	101.4 (10)
**Dz3**	d(CGCACCCAGGCTAGC)	653.6 (65)
	d(CGCACCCAGGCTAG)rC	495.7 (50)
	d(TACAACGACTCTCTCCG)	102.8 (10)
	T^{Bn}-d(ACAACGACTCTCTCCG)	182.2 (18)
	$Napht^{Bn}$-d(ACAACGACTCTCTCCG)	217.8 (22)
	$Napht^{Bn}$-d(TACAACGACTCTCTCCG)	134.3 (13)
**Dz4**	d(CGCACCAGGCTAGCTACAACGAC)	257.9 (26)
	d(CGCACCCAGGCTAGCTACAACGA)rC	68.6 (7)
	d(TCTCTCCG)	720.2 (72)
	T^{Bn}-d(CTCTCCG)	280.3 (28)
	Ph^{Bn}-d(CTCTCCG)	242.7 (24)
	$Napht^{Bn}$-d(CTCTCCG)	229.1 (23)
	Ph^{Bn}-d(TCTCTCCG)	235.1 (24)
	$Napht^{Bn}$-d(TCTCTCCG)	169.6 (17)
**Dz5**	d(CGCACCCAGGCTAGCTACAACGACTC)	118.0 (12)
	d(CGCACCCAGGCTAGCTACAACGACT)rC	66.8 (7)
	d(TCTCCG)	461.8 (46)
	T^{Bn}-d(CTCCG)	207.5 (21)
	Ph^{Bn}-d(CTCCG)	382.1 (38)
	$Napht^{Bn}$-d(CTCCG)	132.1 (13)
	Ph^{Bn}-d(TCTCCG)	344.4 (34)
	$Napht^{Bn}$-d(TCTCCG)	132.1 (13)
**Dz6**	d(CGCACCCAGGCTAGCTACAACGACTCTC)	163.4 (16)
	d(CGCACCCAGGCTAGCTACAACGACTCT)rC	63.7 (6)
	d(TCCG)	720.0 (72)
	T^{Bn}-d(CCG)	623.6 (62)
	Ph^{Bn}-d(CCG)	220.5 (22)
	$Napht^{Bn}$-d(CCG)	106.5 (11)
	Ph^{Bn}-d(TCCG)	308.6 (31)
	$Napht^{Bn}$-d(TCCG)	120.0 (12)

[a] T^{Bn}, $Napht^{Bn}$ and Ph^{Bn} refer respectively a borono-thymidine, a naphthaleneboronic acid or a phenylboronic acid moiety linked to the 5'-extremity. [b] Isolated yields based on 1 µmol scale. [c] RNA synthesis performed with 2'-O-TBDMS protected building blocks.[3] [d] Isolated yields based on 6 nmol scale.

Table 3: *m/z* obtained by MALDI-TOF analysis for pure ONs.

Name	Sequence 5' → 3' [a]	*m/z* calcd[b]	*m/z* found[b]
**Substrate**	r(GGAGAGAGAUGGGUGCG)-$ATTO_{680}$[c]	6389.42[d]	6380.20[d]
**Dz1**	d(CGCACCCAGGCTAGCTACAACGACTCTCTCCG)	9674.23	9676.84
**Dz2**	d(CGCACCCAGGC)	3286.16	3286.25
	d(CGCACCCAGG)rC	3302.15	3302.34
	d(TAGCTACAACGACTCTCTCCG)	6325.10	6325.70
	**T^{Bn}**-d(AGCTACAACGACTCTCTCCG)	6330.92[e]	6326.05[e]
**Dz3**	d(CGCACCCAGGCTAGC)	4521.94	4521.68
	d(CGCACCCAGGCTAG)rC	4537.94	4537.16
	d(TACAACGACTCTCTCCG)	5089.31	5089.01
	**T^{Bn}**-d(ACAACGACTCTCTCCG)	5095.13[e]	5099.60[e]
	**$Napht^{Bn}$**-d(ACAACGACTCTCTCCG)	4998.03[e], 5016.05[f], 5034.07	5000.04[e], 5018.80[f], 5034.40
	**$Napht^{Bn}$**-d(TACAACGACTCTCTCCG)	5302.23[e], 5338.2, 5494.35[g]	5303.83[e], 5338.22, 5494.43[g]
**Dz4**	d(CGCACCAGGCTAGCTACAACGAC)	7275.72	7275.51
	d(CGCACCCAGGCTAGCTACAACGA)rC	7291.72	7291.69
	d(TCTCTCCG)	2335.54	2335.75
	**T^{Bn}**-d(CTCTCCG)	2341.36[e], 2534.48[g]	2343.40[e], 2534.58[g]
	**Ph^{Bn}**-d(CTCTCCG)	2194.20[e], 2230.24	2195.75[e], 2230.64
	**$Napht^{Bn}$**-d(CTCTCCG)	2244.26[e], 2280.29	2245.12[e], 2280.20
	**Ph^{Bn}**-d(TCTCTCCG)	2489.60[h], 2498.40[e], 2516.41[f], 2534.43	2490.81[h], 2500.22[e], 2517.96[f], 2534.94
	**$Napht^{Bn}$**-d(TCTCTCCG)	2552.49[e], 2569.49[f], 2584.49	2552.02[e], 2569.8[f], 2587.04
**Dz5**	d(CGCACCCAGGCTAGCTACAACGACTC)	7869.09	7869.82
	d(CGCACCCAGGCTAGCTACAACGACT)rC	7885.09	7884.81
	d(TCTCCG)	1742.17	1742.83
	**T^{Bn}**-d(CTCCG)	1747.98[e], 1940.10[g]	1749.33[e], 1941.85[g]
	**Ph^{Bn}**-d(CTCCG)	1600.83[e], 1636.86, 1792.95[g]	1603.47[e], 1638.25, 1794.79[g]
	**$Napht^{Bn}$**-d(CTCCG)	1642.09[h]	1643.72[h]
	**Ph^{Bn}**-d(TCTCCG)	1905.02[e], 1941.05	1907.46[e], 1941.42
	**$Napht^{Bn}$**-d(TCTCCG)	1955.08[e], 1991.11	1956.55[e], 1991.69
**Dz6**	d(CGCACCCAGGCTAGCTACAACGACTCTC)	8462.47	8462.99
	d(CGCACCCAGGCTAGCTACAACGACTCT)rC	8478.47	8478.09
	d(TCCG)	1148.80	1148.79
	**T^{Bn}**-d(CCG)	1154.61[e], 1172.62[f], 1191.64	1155.95[e], 1174.10[f], 1191.81
	**Ph^{Bn}**-d(CCG)	998.66[h], 1042.68	998.66[h], 1042.63
	**$Napht^{Bn}$**-d(CCG)	1048.72[h], 1094.55	1050.91[h], 1094.89
	**Ph^{Bn}**-d(TCCG)	1302.85[h], 1346.67	1302.65[h], 1346.91
	**$Napht^{Bn}$**-d(TCCG)	1352.91[h], 1397.74	1353.49[h], 1397.08

[a] T^{Bn}, $Napht^{Bn}$ and Ph^{Bn} refer respectively a borono-thymidine, a naphthaleneboronic acid or a phenylboronic acid moiety linked to the 5'-extremity. [b] *m/z* calculated and found for $[M-H]^-$. [c] RNA synthesis performed with 2'-O-TBDMS residues.[3] [d] *m/z* obtained for $[M+H]^+$. [e] *m/z* calculated and found for $[M-2H_2O-H]^-$. [f] *m/z* calculated and found for $[M-H_2O-H]^-$. [g] *m/z* calculated and found for $[M-2H_2O+citrate-H]^-$. [h] *m/z* calculated and found for $[M-B(OH)_2-H]^-$.

4. Cleavage reactions

Cleavage reactions were carried out under single turnover conditions with a 100-fold excess of DNAzyme over the RNA substrate. The split DNAzyme fragments (2.0 µM, 60.0 pmol) and RNA substrate (20 nM, 0.6 pmol) were mixed in 50µL of Tris-HCl buffer (50 mM, pH 5.5, 8.6 or 9.6), heated for 2 min at 90°C and incubated for 15 min at 25°C. $MgCl_2$ solution (20 mM) was added to start the reaction, which was performed at 25°C. After defined time points (5, 10, 15, 20, 30, 45, 60, 120 min) aliquots of 1 µL were taken and added to 19 µL stop mix (7 M urea, 50 mM EDTA), and kept on ice or stored at -20°C until analysed on a denaturing 15 % polyacrylamide gel using a LICOR-DNA sequencer. Data was processed with Gene ImagIR 4.05 software. The cleaved substrate fraction plotted over time was fitted to the equation :

$$[S]=A(1-e^{-kt})$$

Where [S] represents the fraction of substrate uncleaved at time t and A the percentage of total RNA cleaved after 2 hours of incubation.

5. Gel electrophoresis analysis and time conversion curves

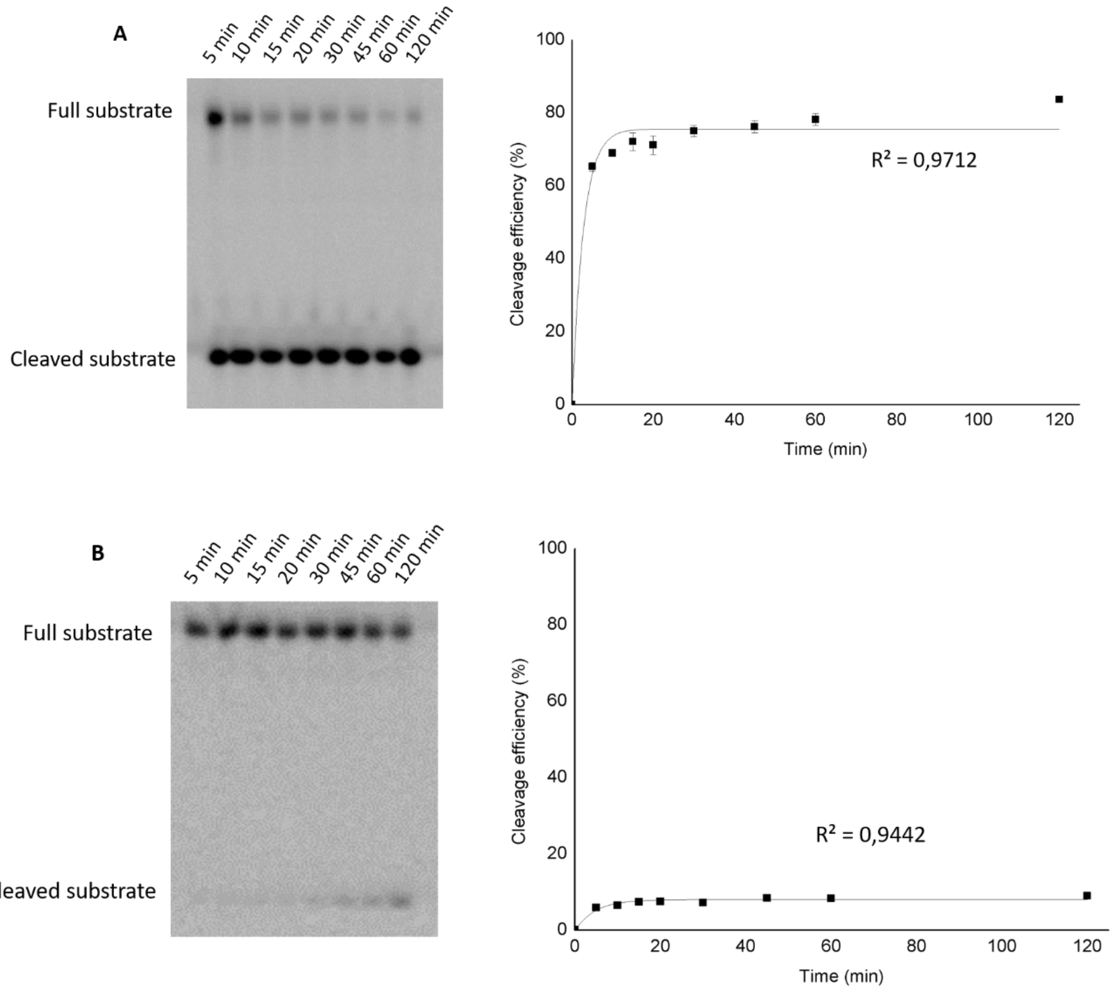

Figure S19. Gel electrophoresis analysis and time conversion curve of **Dz1** (**A**) in presence of 20 mM $MgCl_2$ (Table 1, Entry 1) and (**B**) in the absence of $MgCl_2$.

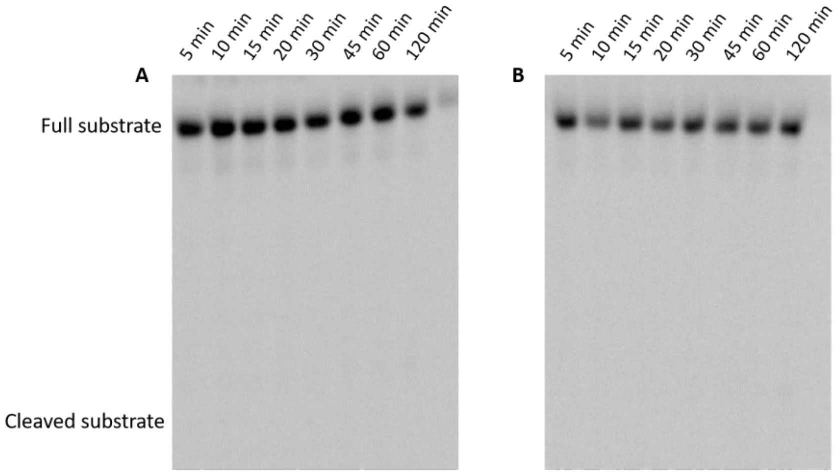

Figure S20. Gel electrophoresis analysis of **Dz2** : (**A**) X=C, Y=T (Table 1, Entry 2) and (**B**) X= $\mathbf{T^{Bn}}$, Y= rC (Table 1, Entry 3).

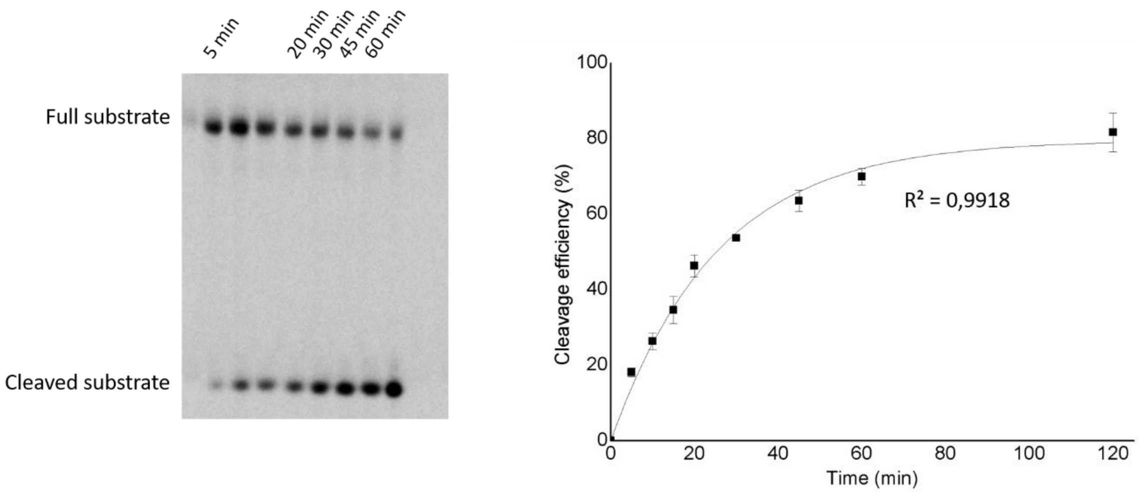

Figure S21. Gel electrophoresis analysis and time conversion curve of **Dz3** (X=T, Y=C) (Table 1, Entry 4).

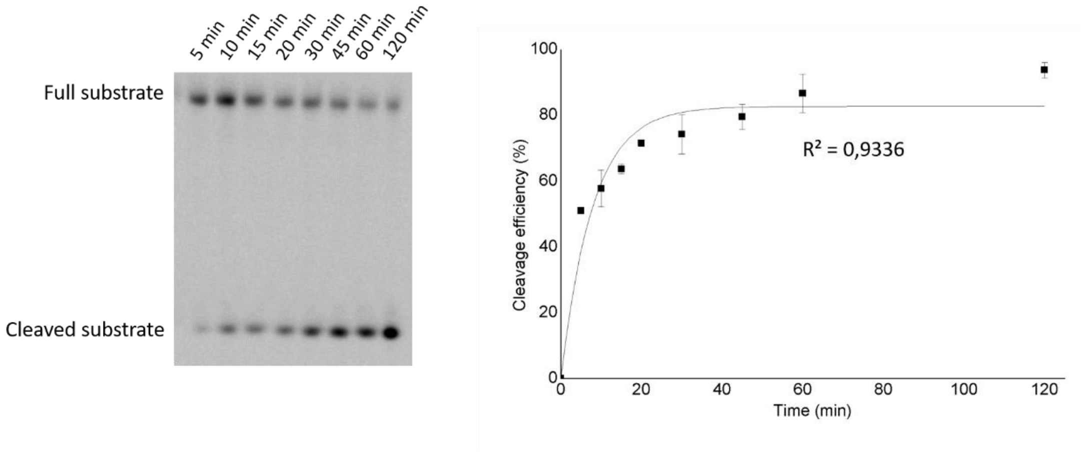

Figure S22. Gel electrophoresis analysis and time conversion curve of **Dz3** (X=$\mathbf{T^{bn}}$, Y=rC) (Table 1, Entry 5).

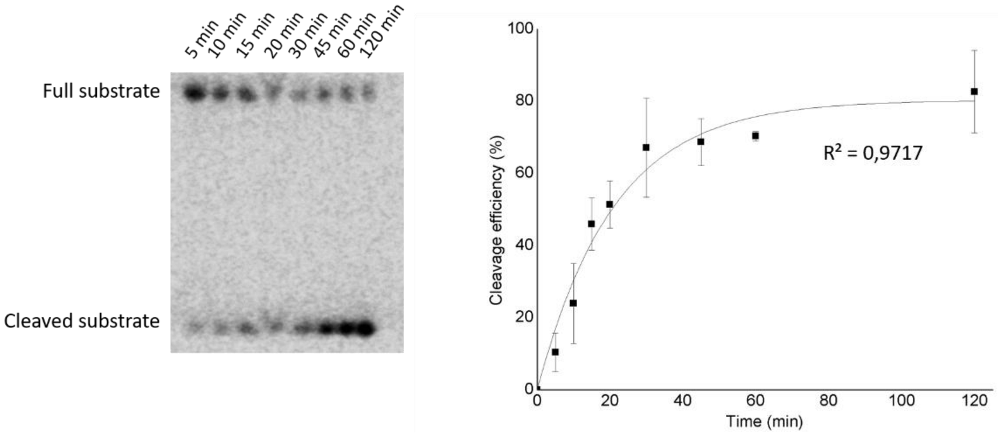

Figure S23. Gel electrophoresis analysis and time conversion curve of **Dz3** (X=$\mathbf{T^{bn}}$, Y=C) (Table 1, Entry 6).

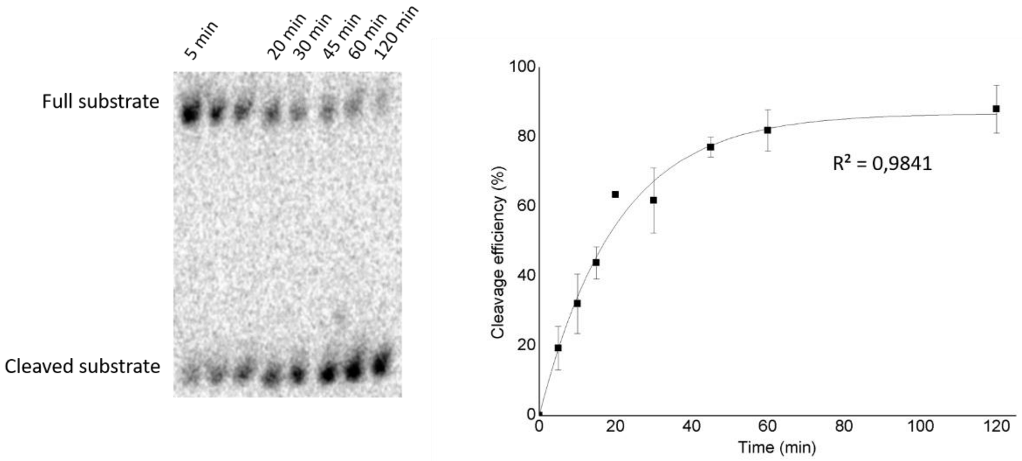

Figure S24. Gel electrophoresis analysis and time conversion curve of **Dz3** (X=**Naphtbn**, Y=rC) (Table 1, Entry 7).

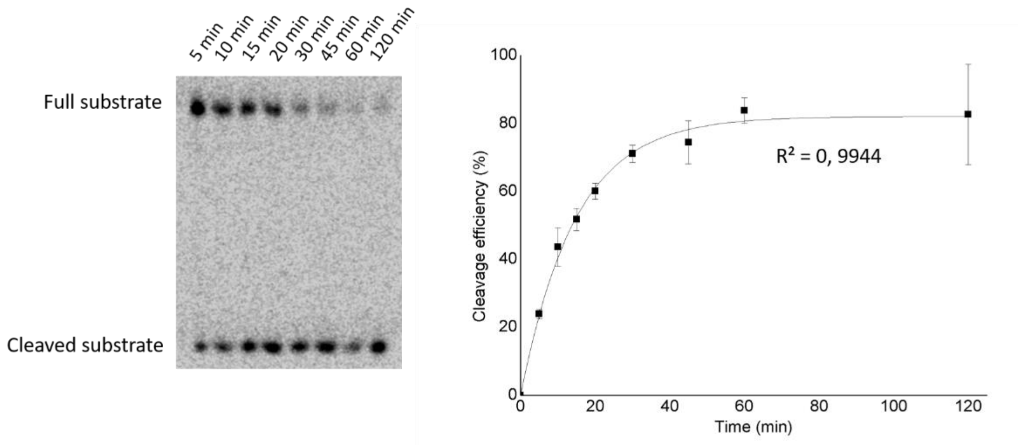

Figure S25. Gel electrophoresis analysis and time conversion curve of **Dz3** (X=T**Naphtbn**, Y=rC) (Table 1, Entry 8).

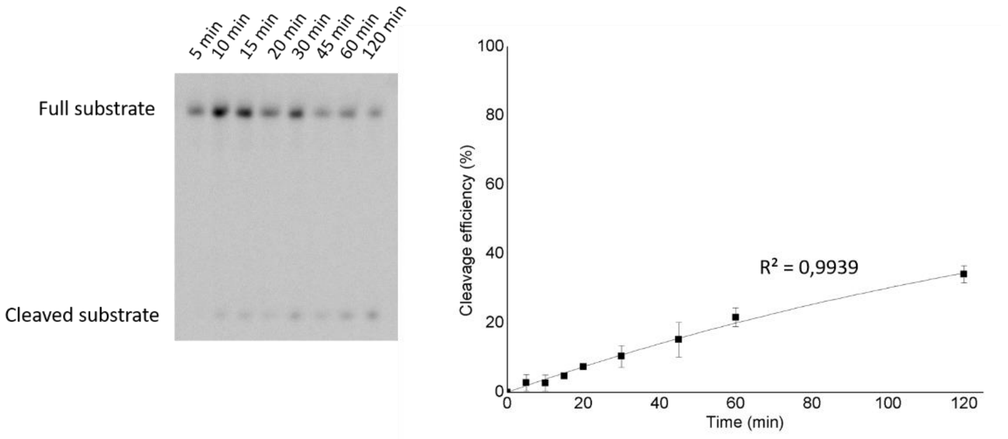

Figure S26. Gel electrophoresis analysis and time conversion curve of **Dz4** (X=T, Y=C) (Table 1, Entry 9).

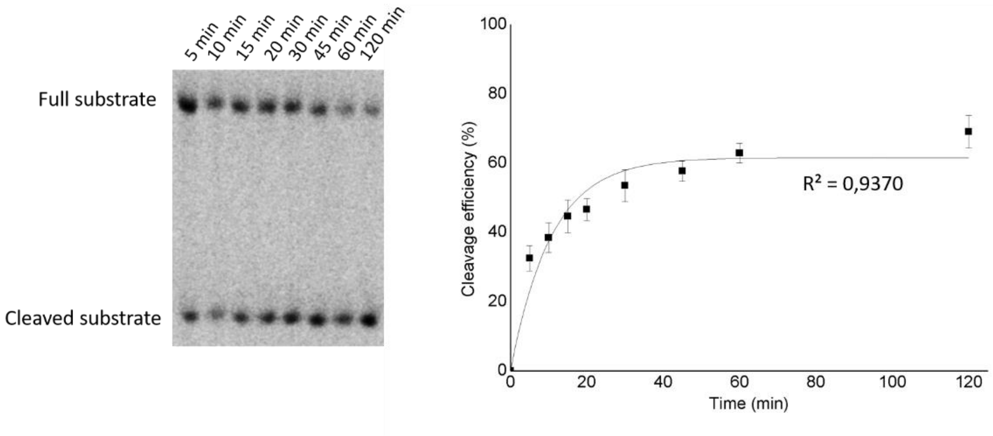

Figure S27. Gel electrophoresis analysis and time conversion curve of **Dz4** (X=**T^{bn}**, Y=rC) (Table 1, Entry 10).

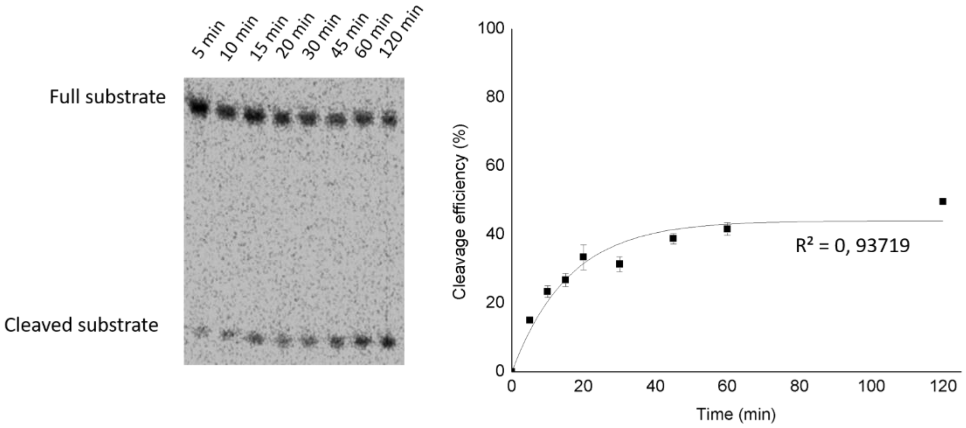

Figure S28. Gel electrophoresis analysis and time conversion curve of **Dz4** (X=**Ph^{bn}**, Y=rC) (Table 1, Entry 11).

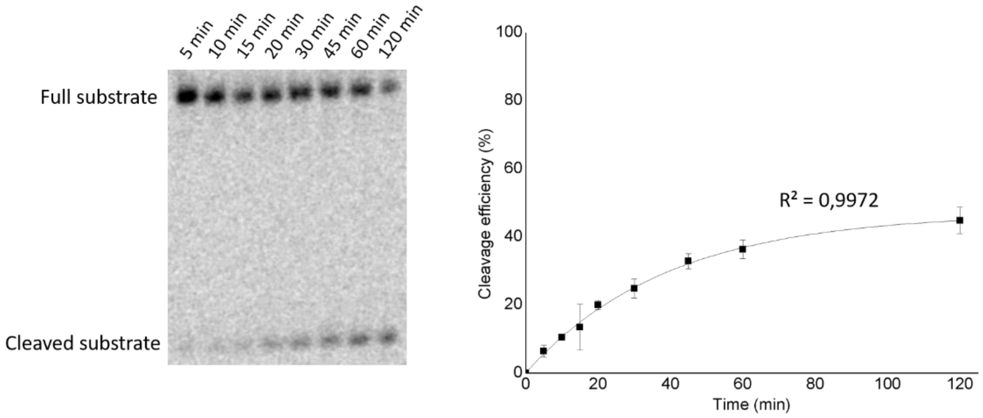

Figure S29. Gel electrophoresis analysis and time conversion curve of **Dz4** (X=**Napht^{bn}**, Y=rC) (Table 1, Entry 12).

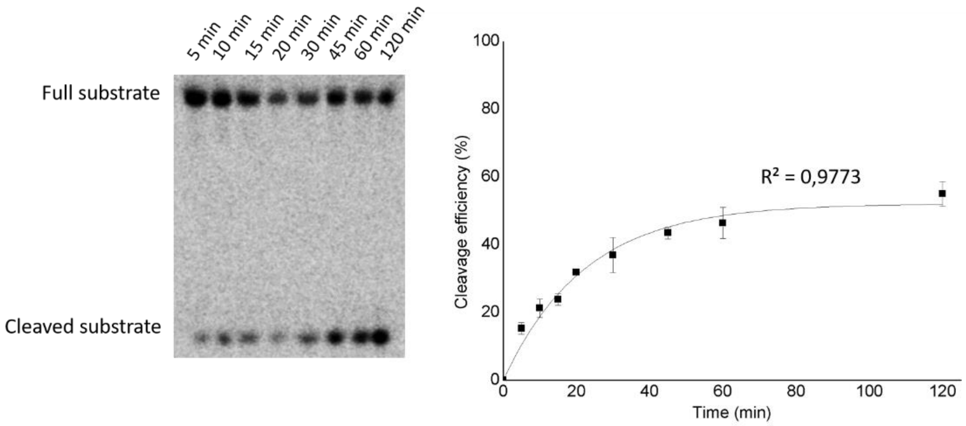

Figure S30. Gel electrophoresis analysis and time conversion curve of **Dz4** (X=T**Ph**^{bn}, Y=rC) (Table 1, Entry 13).

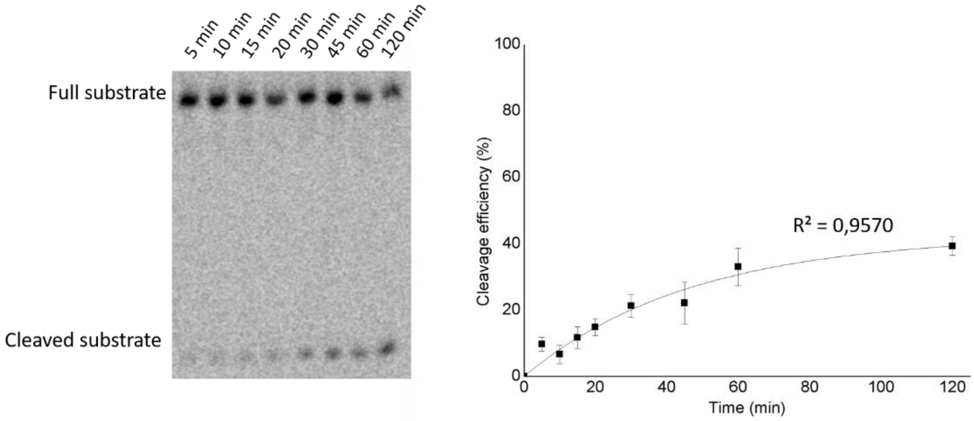

Figure S31. Gel electrophoresis analysis and time conversion curve of **Dz4** (X=T**Napht**^{bn}, Y=rC) (Table 1, Entry 14).

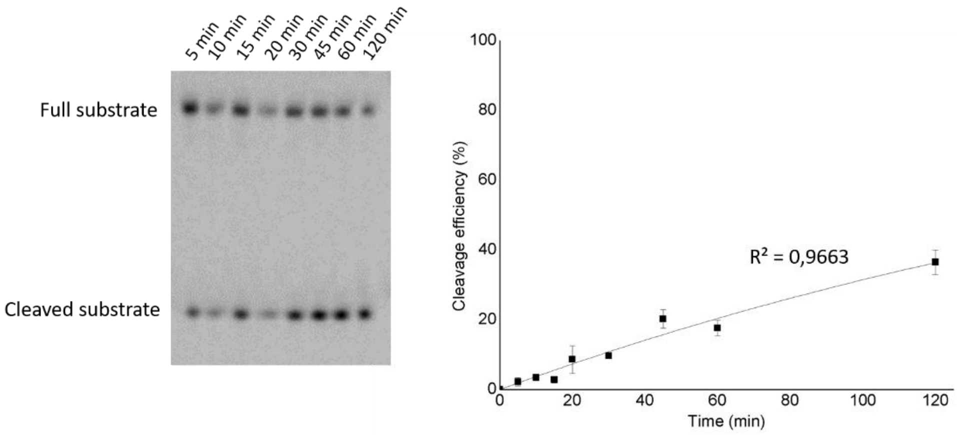

Figure S32. Gel electrophoresis analysis and time conversion curve of **Dz5** (X=T, Y=rC) (Table 1, Entry 15).

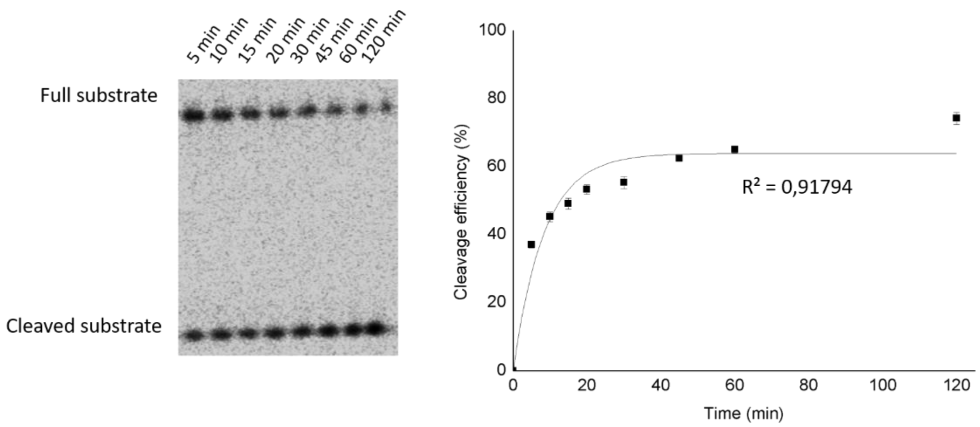

Figure S33. Gel electrophoresis analysis and time conversion curve of **Dz5** (X=**T**^{bn}, Y=rC) (Table 1, Entry 16).

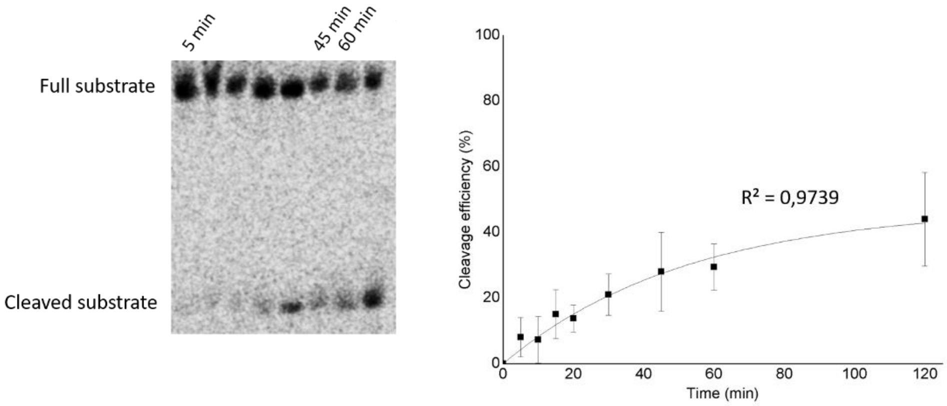

Figure S34. Gel electrophoresis analysis and time conversion curve of **Dz5** (X=**Ph**^{bn}, Y=rC) (Table 1, Entry 17).

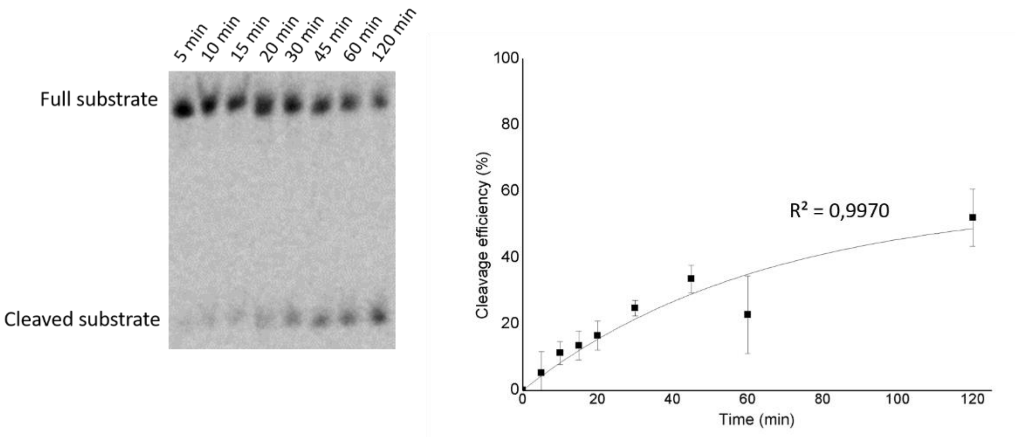

Figure S35. Gel electrophoresis analysis and time conversion curve of **Dz5** (X=**Napht**^{bn}, Y=rC) (Table 1, Entry 18).

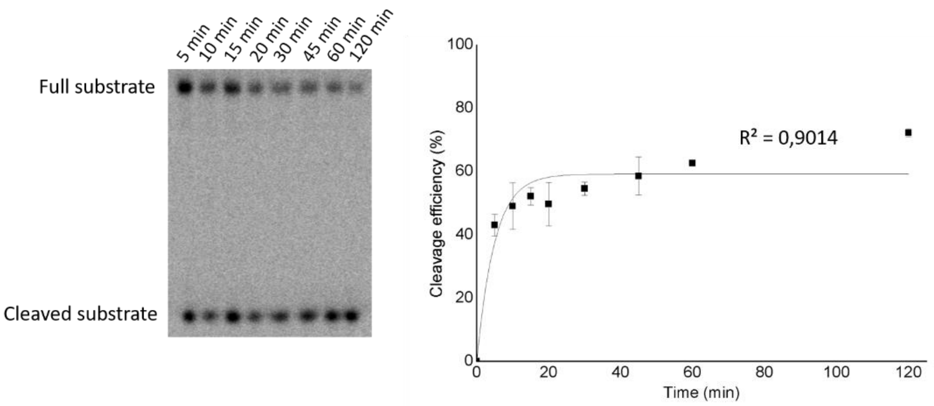

Figure S36. Gel electrophoresis analysis and time conversion curve of **Dz5** (X=T**Ph^{bn}**, Y=rC) (Table 1, Entry 19).

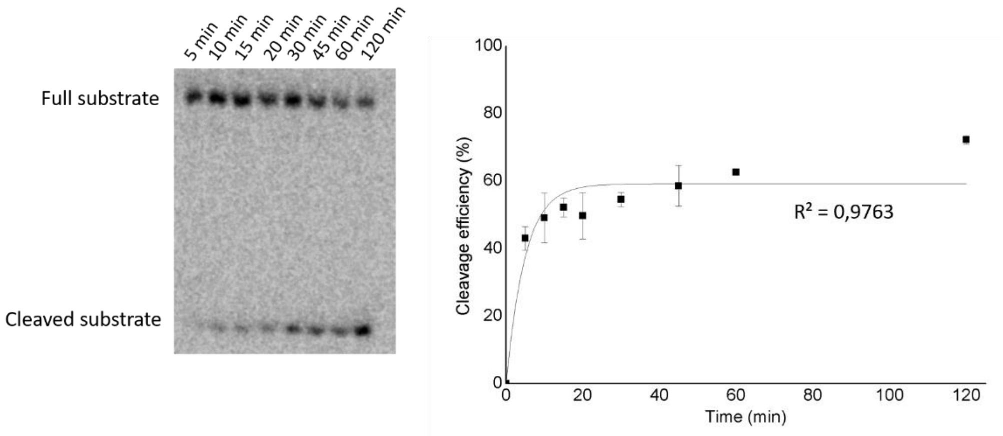

Figure S37. Gel electrophoresis analysis and time conversion curve of **Dz5** (X=T**Napht^{bn}**, Y=rC) (Table 1, Entry 20).

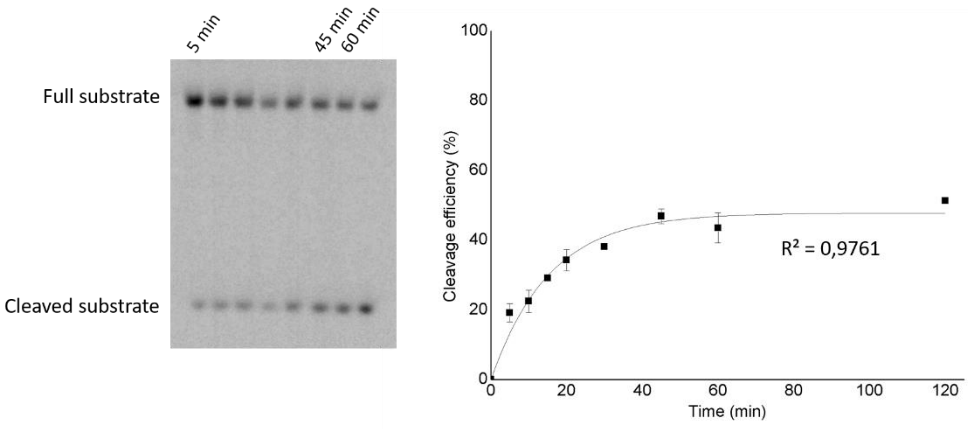

Figure S38. Gel electrophoresis analysis and time conversion curve of **Dz6** (X=T, Y=rC) (Table 1, Entry 21).

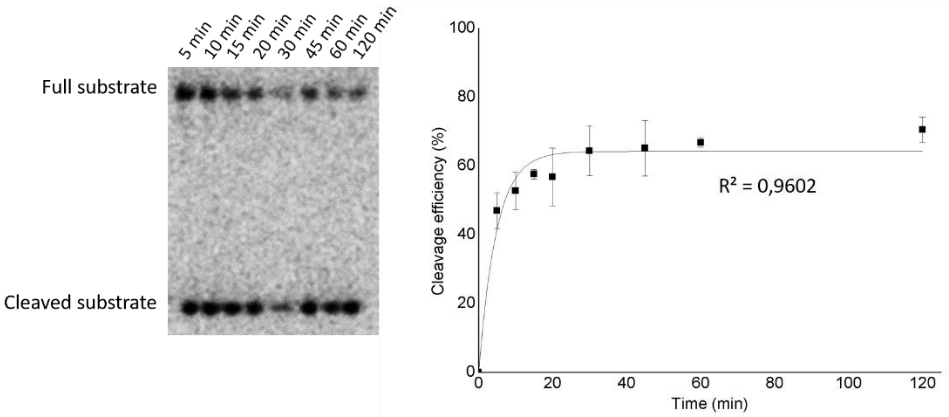

Figure S39. Gel electrophoresis analysis and time conversion curve of **Dz6** (X=**T^{bn}**, Y=rC) (Table 1, Entry 22).

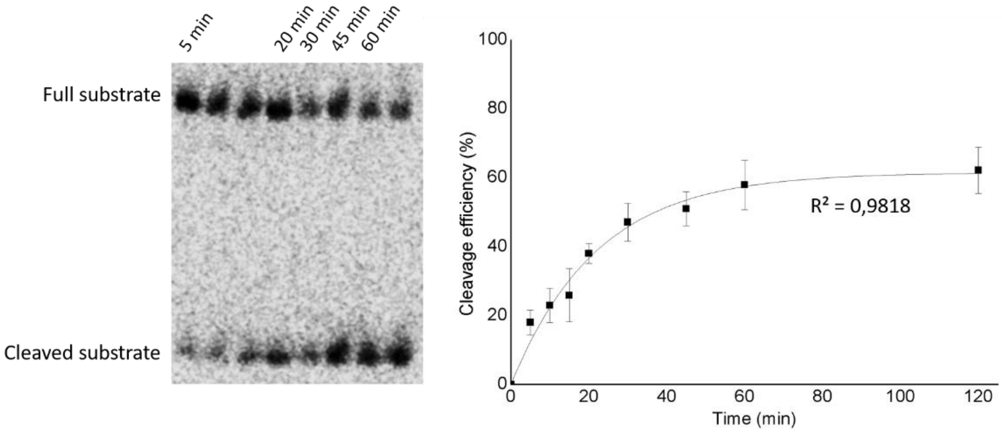

Figure S40. Gel electrophoresis analysis and time conversion curve of **Dz6** (X=**Ph^{bn}**, Y=rC) (Table 1, Entry 23).

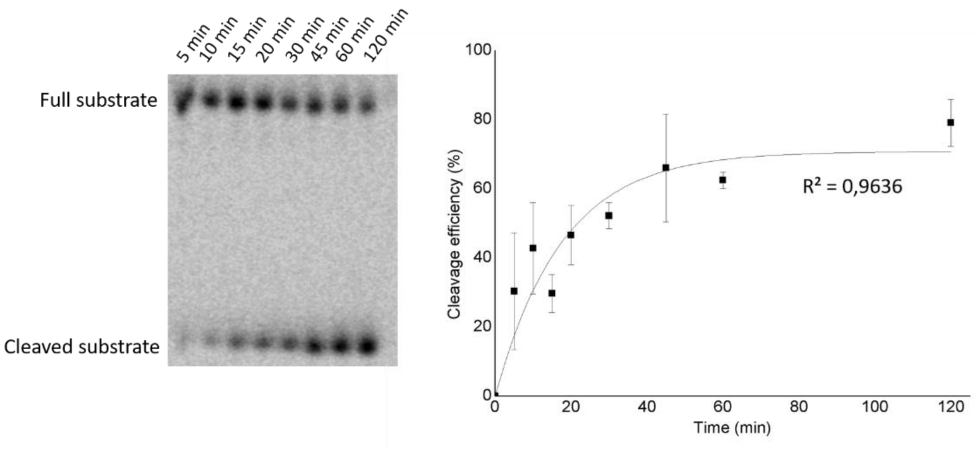

Figure S41. Gel electrophoresis analysis and time conversion curve of **Dz6** (X=**Napht^{bn}**, Y=rC) (Table 1, Entry 24).

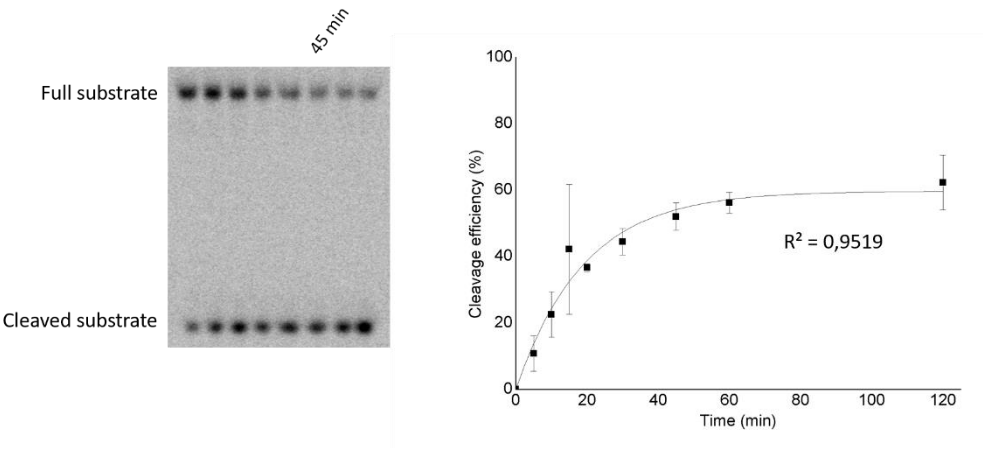

Figure S42. Gel electrophoresis analysis and time conversion curve of **Dz6** (X=T**Ph**^{bn}, Y=rC) (Table 1, Entry 25).

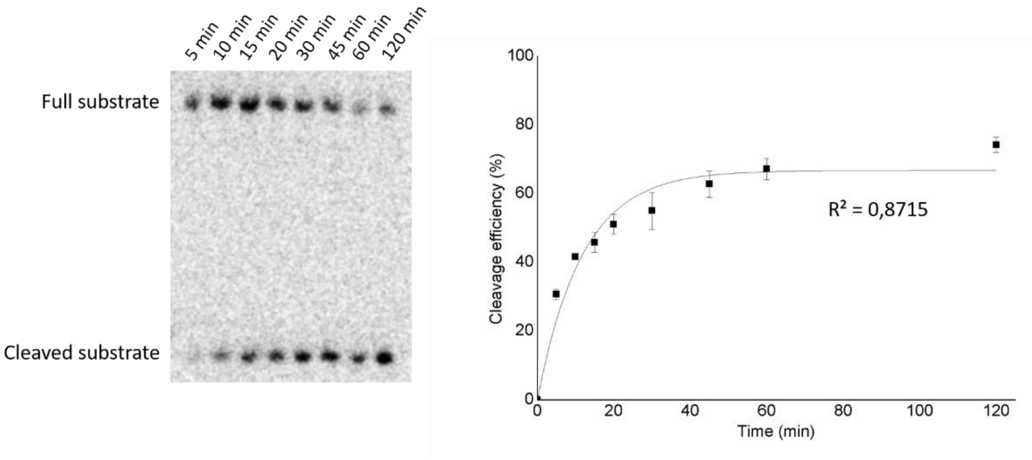

Figure S43. Gel electrophoresis analysis and time conversion curve of **Dz6** (X=T**Napht**^{bn}, Y=rC) (Table 1, Entry 26).

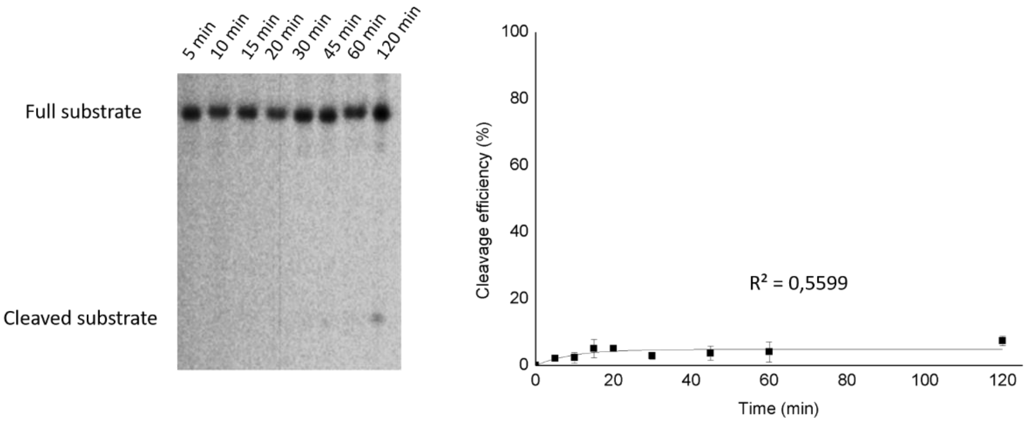

Figure S44: Gel electrophoresis analysis and time conversion curve of **Dz1** at pH 5.5 (k = 0.106 ± 0.062 min^{-1} and A = 4.9 ± 0.8 %).

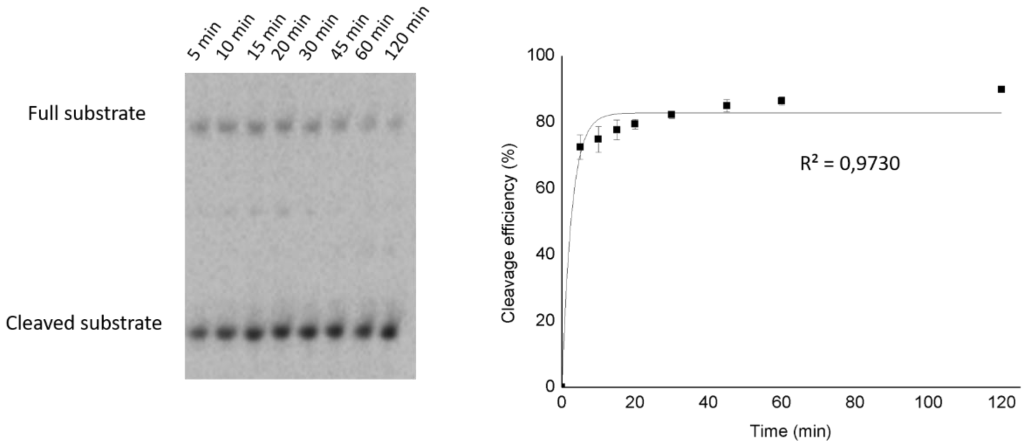

Figure S45: Gel electrophoresis analysis and time conversion curve of **Dz1** at pH 9.6 (k = 0.378 ± 0.076 min^{-1} and A = 82.8 ± 1.8 %).

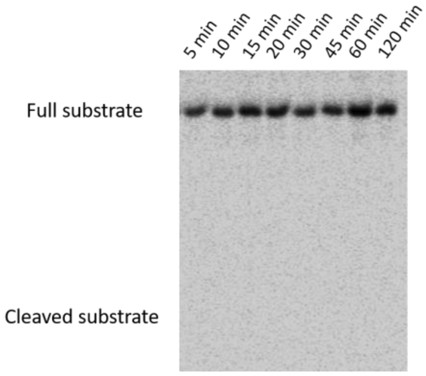

Figure S46: Gel electrophoresis analysis of **Dz4** (X=T, Y=C) at pH 5.5.

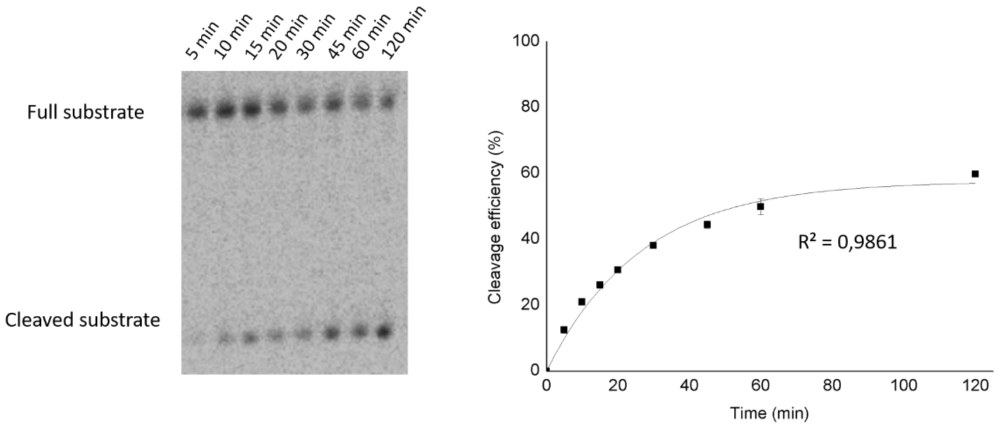

Figure S47: Gel electrophoresis analysis and time conversion curve of **Dz4** (X=T, Y=C) at pH 9.6 (k = 0.038 ± 0.003 min^{-1} and A = 57.6 ± 2.1 %).

BIBLIOGRAPHIE

[1] D. Luvino, C. Baraguey, M. Smietana, J.-J. Vasseur, *Chemical Communications* **2008**, 2352.

[2] A. Gimenez Molina, I. Barvik, S. Müller, J.-J. Vasseur, M. Smietana, *Organic & Biomolecular Chemistry* **2018**, *16*, 8824–8830.

[3] T. Lavergne, J.-R. Bertrand, J.-J. Vasseur, F. Debart, *Chemistry – A European Journal* **2008**, *14*, 9135–9138.
